# Development of a Dual-Fluorescent-Reporter System in Clostridioides difficile Reveals a Division of Labor between Virulence and Transmission Gene Expression

**DOI:** 10.1128/msphere.00132-22

**Published:** 2022-05-31

**Authors:** M. Lauren Donnelly, Shailab Shrestha, John W. Ribis, Pola Kuhn, Maria Krasilnikov, Carolina Alves Feliciano, Aimee Shen

**Affiliations:** a Department of Molecular Biology and Microbiology, Tufts University School of Medicine, Boston, Massachusetts, USA; b Graduate School of Biomedical Sciences, Tufts University School of Medicine, Boston, Massachusetts, USA; University of Iowa

**Keywords:** *Clostridioides difficile*, division of labor, fluorescent reporter, sporulation, toxin, transcriptional reporter

## Abstract

The bacterial pathogen Clostridioides difficile causes gastroenteritis by producing toxins and transmits disease by making resistant spores. Toxin and spore production are energy-expensive processes that are regulated by multiple transcription factors in response to many environmental inputs. While toxin and sporulation genes are both induced in only a subset of C. difficile cells, the relationship between these two subpopulations remains unclear. To address whether C. difficile coordinates the generation of these subpopulations, we developed a dual-transcriptional-reporter system that allows toxin and sporulation gene expression to be simultaneously visualized at the single-cell level using chromosomally encoded mScarlet and mNeonGreen fluorescent transcriptional reporters. We then adapted an automated image analysis pipeline to quantify toxin and sporulation gene expression in thousands of individual cells under different medium conditions and in different genetic backgrounds. These analyses revealed that toxin and sporulation gene expression rarely overlap during growth on agar plates, whereas broth culture increases this overlap. Our results suggest that certain growth conditions promote a “division of labor” between transmission and virulence gene expression, highlighting how environmental inputs influence these subpopulations. Our data further suggest that the RstA transcriptional regulator skews the population to activate sporulation genes rather than toxin genes. Given that recent work has revealed population-wide heterogeneity for numerous cellular processes in C. difficile, we anticipate that our dual-reporter system will be broadly useful for determining the overlap between these subpopulations.

**IMPORTANCE** Clostridioides difficile is an important nosocomial pathogen that causes severe diarrhea by producing toxins and transmits disease by producing spores. While both processes are crucial for C. difficile disease, only a subset of cells express toxins and/or undergo sporulation. Whether C. difficile coordinates the subset of cells inducing these energy-expensive processes remains unknown. To address this question, we developed a dual-fluorescent-reporter system coupled with an automated image analysis pipeline to rapidly compare the expression of two genes of interest across thousands of cells. Using this system, we discovered that certain growth conditions, particularly growth on agar plates, induce a “division of labor” between toxin and sporulation gene expression. Since C. difficile exhibits phenotypic heterogeneity for numerous vital cellular processes, this novel dual-reporter system will enable future studies aimed at understanding how C. difficile coordinates various subpopulations throughout its infectious disease cycle.

## INTRODUCTION

Clostridioides difficile is a Gram-positive, spore-forming, anaerobic pathogen that is the leading cause of nosocomial diarrhea worldwide and health care-associated infections in the United States (1, 2). C. difficile infections typically occur in patients whose normal colonic microflora is disrupted, such as individuals who have undergone antimicrobial therapy (1). C. difficile infections often disseminate in health care settings because this organism’s resistant, infectious, aerotolerant spore morphotype is transmitted by the fecal-oral route and can persist in the environment for long periods (3–6). When C. difficile spores are ingested, they germinate in the guts of dysbiotic hosts in response to specific bile acids (7) and outgrow into vegetative cells. These cells produce the glucosylating toxins toxin A (TcdA) and toxin B (TcdB), which are responsible for C. difficile disease symptoms (8). By glucosylating Rho family GTPases, the toxins induce actin cytoskeleton collapse and loss of tight junctions in the colon (8). The resulting damage to the colonic epithelium elicits a massive host inflammatory response, which can lead to pseudomembranous colitis, colonic perforation, toxic megacolon, and even death (9).

Toxin production and spore formation are frequently coordinated in spore-forming bacteria, with toxin production being induced during early stages of sporulation in pathogens like Clostridium perfringens and some strains of Clostridium botulinum and Bacillus thuringiensis (10–15). This temporal order likely enhances the transmission of these pathogens because the toxins induce diarrhea or death of the host to promote dissemination of the spores into the environment (10, 16). However, the relationship between toxin and sporulation genes is often strain dependent. For example, some B. thuringiensis strains generate a “division of labor” in which some cells within the population express toxin genes and a different subset induce sporulation (16, 17). In C. difficile, recent work has shown that both toxin and sporulation genes are heterogeneously expressed within a given population, with only a subset of cells expressing toxin genes (18) and a subset expressing sporulation genes (19). While these two subpopulations can overlap (18), it is unclear how frequently this occurs on a population-wide level.

Toxin and sporulation gene expression in C. difficile is controlled by numerous environmental signals. For example, both sets of genes are repressed by glucose and preferentially induced during stationary phase in C. difficile, suggesting that these processes are induced by nutrient starvation (20). The levels of cyclic di-GMP (c-di-GMP), autoinducing peptides, and other signaling molecules also modulate these processes (21, 22). Environmental control of toxin and sporulation gene expression is mediated by a complex network of overlapping genetic circuits in C. difficile. *tcdA* and *tcdB* toxin gene expression is activated by the alternative sigma factor TcdR and is bistable in many C. difficile strains. Bistable expression of *tcdA* is a function of TcdR’s autoregulation: TcdR enhances its production through a positive feedback loop, and breaking this positive feedback loop through the inducible expression of *tcdR* results in *tcdA* being uniformly expressed (i.e., with a unimodal rather than bimodal distribution) (18). In addition to TcdR, several transcription factors regulate *tcdR* expression in response to nutrient availability and growth phase (23).

Sporulation is controlled by the master transcriptional activator, Spo0A, which initiates sporulation by inducing the expression of genes encoding sporulation-specific sigma factors and genes whose products activate these sigma factors, which subsequently drive sporulation (24). *spo0A* transcription and/or Spo0A activity is controlled by several of the same regulatory factors that affect toxin production (21, 25–27). For example, CodY is a global regulator that represses both sporulation and toxin gene expression (28) when branched-chain amino acids and GTP are abundant (28–31). In addition, the carbon catabolite protein A (CcpA) represses toxin and sporulation gene expression in the presence of glucose and other carbohydrates (32). SigH is a positive regulator of sporulation but a negative regulator of toxin production (33), while the bifunctional transcription factor RstA represses toxin production but enhances sporulation. RstA directly inhibits transcription of *tcdA*, *tcdB*, and *tcdR* (34) while indirectly promoting the expression sporulation genes (35).

Interestingly, some studies suggest that toxin and sporulation gene expression may be coordinated in C. difficile because cross talk between TcdR and Spo0A function has been reported (25, 36, 37). For example, Spo0A negatively regulates toxin production in some but not all isolates of C. difficile, likely through indirect mechanisms (37) and loss of toxin production due to mutation of *tcdR* enhances sporulation in some strain backgrounds (36). Notably, many of these findings are strain specific as well as highly dependent on growth and medium conditions, leading to conflicting reports of the relationship between sporulation and toxin production (38).

Disentangling how C. difficile coordinates toxin and sporulation gene expression requires the development of transcriptional reporters that can be simultaneously visualized in individual cells. In this study, we optimized a chromosomally encoded dual-reporter system for use in C. difficile that overcomes C. difficile’s high intrinsic autofluorescence (39). Using these reporters, we addressed whether C. difficile coordinates toxin and sporulation gene expression under different medium conditions and in different mutant backgrounds. Our results suggest that certain growth conditions promote a division of labor between toxin and sporulation gene expression, with minimal overlap between the subsets of cells expressing toxin and sporulation genes. Given that recent studies have identified several genes beyond toxin and sporulation genes with bimodal patterns of expression (40–42), our novel dual-reporter system should be broadly useful for studying how these different subpopulations of C. difficile cells overlap and how nutritional and environmental inputs affect this overlap.

## RESULTS

### Development of chromosomally encoded dual-color transcriptional reporters for use in C. difficile.

To visualize toxin and sporulation gene expression simultaneously, we first needed to identify reporters with nonoverlapping spectra that were bright enough to overcome C. difficile’s autofluorescence in the green channel (39, 43). This autofluorescence has traditionally limited the utility of green-emitting reporters like green fluorescent protein (GFP) and the flavin mononucleotide (FMN)-based reporter phiLOV to genes that are highly expressed (39, 44). However, mNeonGreen (mNG) is a rapidly maturing, yellow-green monomeric fluorescent protein made by Branchiostoma lanceolatum that is ~4-fold brighter than GFP (45, 46), so we hypothesized that it might be bright enough to use as a fluorescent transcriptional reporter. In addition, mNeonGreen’s spectral properties are closer to that of yellow fluorescent protein (YFP), reducing overlap with C. difficile’s autofluorescent signal. Finally, mNeonGreen is spectrally compatible with mScarlet (mSc), a derivative of mCherry that is ~3-fold brighter than mCherry (47), such that mNeonGreen and mScarlet can be used in dual-transcriptional-reporter and protein localization systems (46, 48).

**FIG 1:**
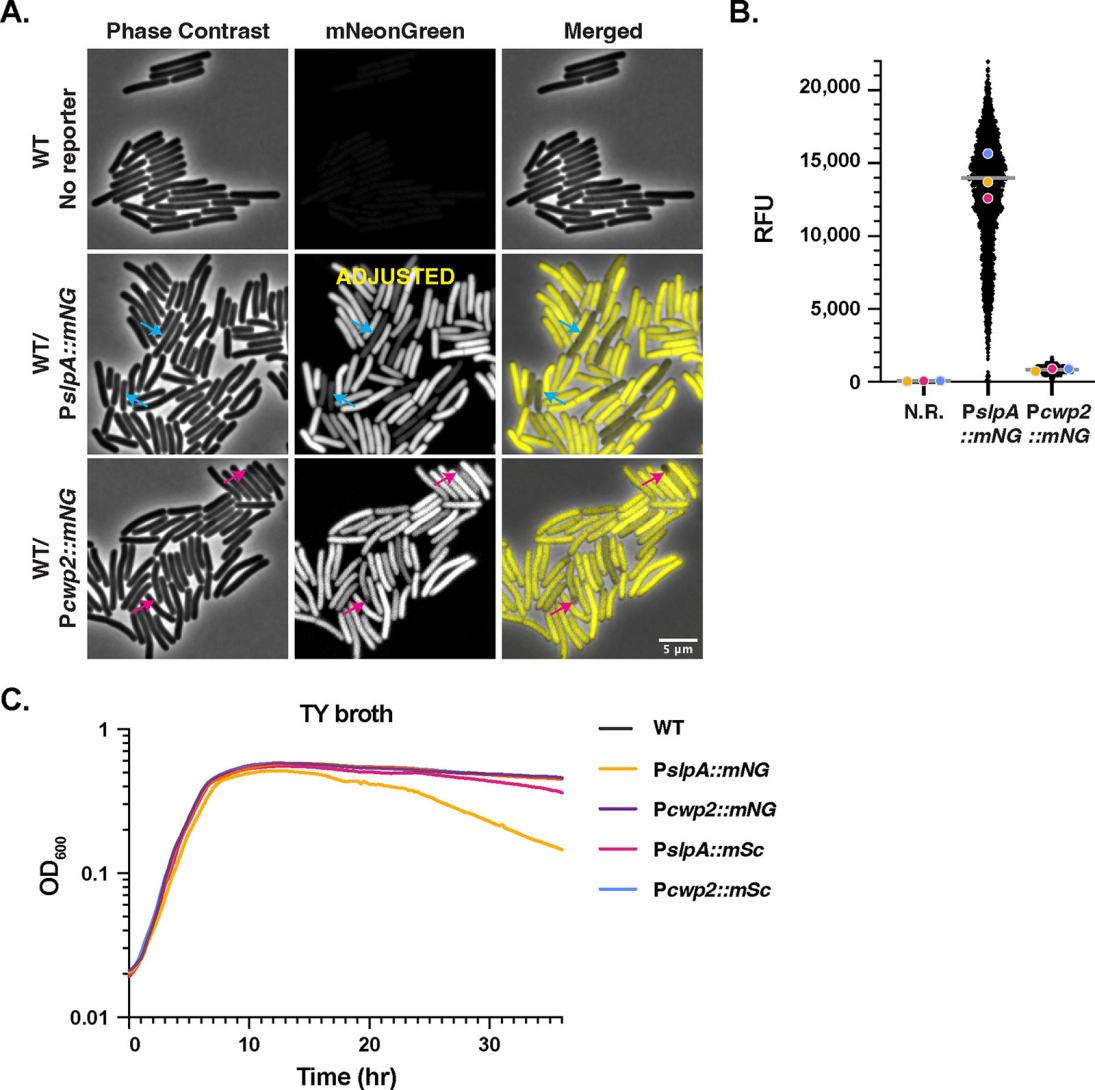
Identification of constitutive *mNeonGreen* reporters that overcome C. difficile’s autofluorescence. (A) Fluorescence microscopy analyses of live bacterial cells showing wild-type 630Δ*erm* (no reporter) and 630Δ*erm* carrying *mNeonGreen* coupled to either the *slpA* or *cwp2* promoters after overnight growth in TY media. Phase-contrast microscopy was used to visualize the cells. Blue arrows highlight cells where the mNeonGreen signal appears reduced relative to that in other cells in the population for the P*slpA*::*mNG* strain. Pink arrows highlight lower levels of mNeonGreen signal in the forespores of cells undergoing sporulation. The merge of phase-contrast images and images with mNeonGreen pseudocolored in yellow is shown. The P*slpA*::*mNG* signal was adjusted for brightness/contrast because this reporter is ~10-fold brighter than P*cwp2*::*mNG*. (B) SuperSegger-based quantification (55) of the mean fluorescence intensity for each cell is shown as a black dot on the scatterplot. The magenta, yellow, and blue dots represent the median fluorescence intensities for the three biological replicates. The gray line represents the mean fluorescence value for each reporter based on the average of the three biological replicate’s median value (80). N.R., wild type with no reporter. (C) OD_600_ growth curve of the indicated strains during growth in TY broth. The graph shown is for a single biological replicate that is representative of three biological replicates.

**FIG 2:**
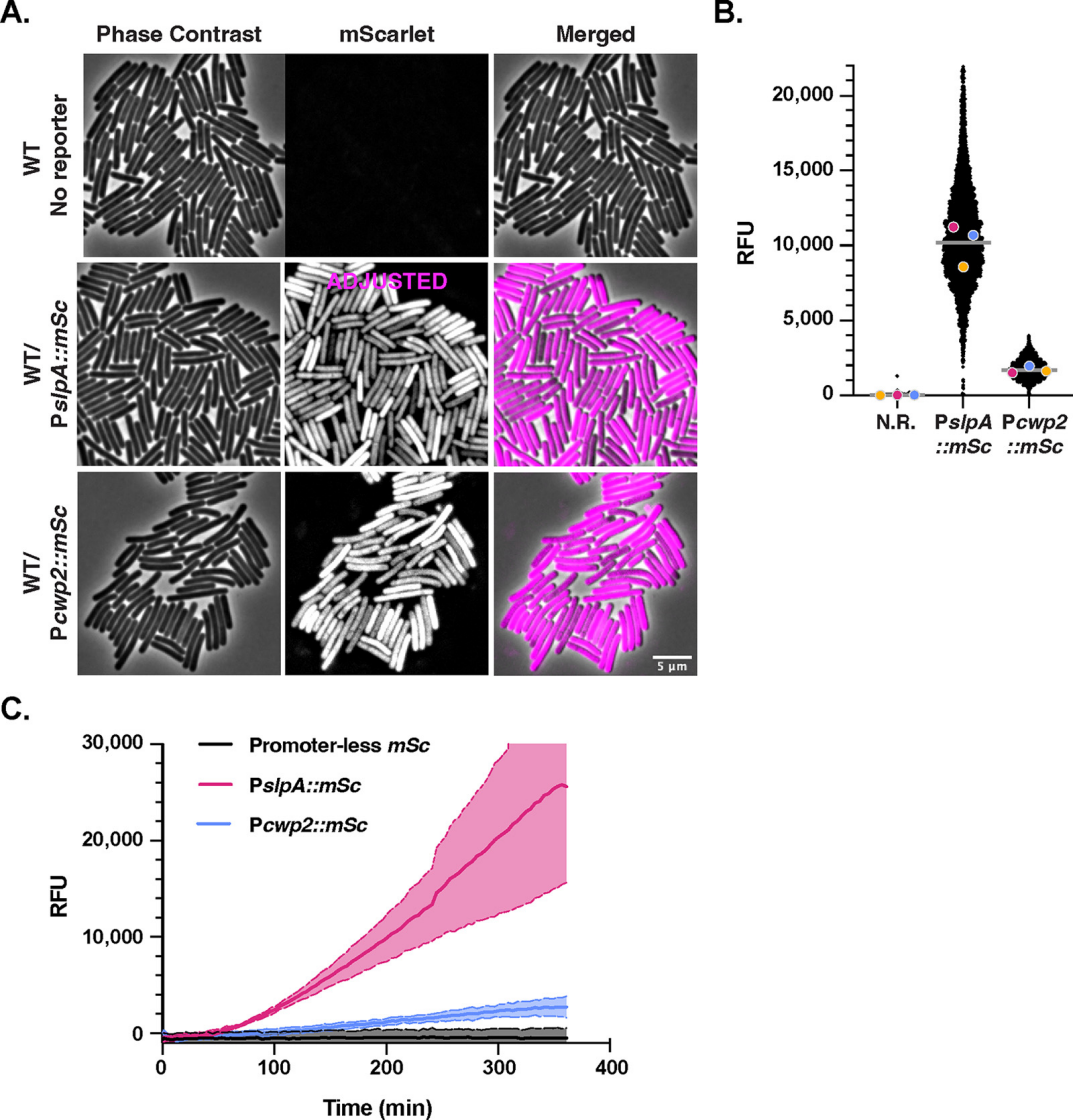
Optimization of constitutive mScarlet reporters for use in C. difficile. (A) Fluorescence microscopy analyses of fixed cells for wild-type 630Δ*erm* (no reporter) and 630Δ*erm* carrying *mScarlet* coupled to either the *slpA* or *cwp2* constitutive promoters after overnight growth in TY broth. Phase-contrast microscopy was used to visualize all bacterial cells. The merge of phase-contrast images and images with mScarlet pseudocolored in magenta is shown. The P*slpA*::*mSc* signal was adjusted for brightness/contrast because this reporter is much brighter than P*cwp2*::*mSc*. (B) SuperSegger-based quantification of the mean fluorescence intensity for each cell is shown as a black dot on the scatterplot. The magenta, yellow and blue dots represent the median fluorescence intensities for the three biological replicates. The gray line represents the mean fluorescence value for each reporter based on the average of the two biological replicates’ median value (80). N.R., wild type with no reporter. (C) Fluorescence intensities of overnight TY medium cultures of the indicated strains after exposure to oxygen over the course of 36 h. Data derive from three biological replicates.

To test whether mNeonGreen and/or mScarlet fluorescent reporters would be bright enough for use in C. difficile, we codon optimized *mNeonGreen* and *mScarlet* and coupled these genes to two different constitutive promoters: P*slpA* and P*cwp2*. *slpA* encodes the major S layer protein and is the most highly transcribed gene in our prior transcriptome sequencing (RNA-Seq) analyses in C. difficile (49–51), while *cwp2* encodes a cell wall protein (52), and its promoter has previously been used as a constitutive promoter (53). The promoters were followed by the ribosome binding site of the *slpA* gene, and the resulting constructs were integrated in single copy into the *pyrE* locus of 630Δ*erm*Δ*pyrE* using allele-coupled exchange (54). After the resulting strains had been grown overnight in tryptone yeast (TY) medium, we spotted the cultures onto agarose pads under ambient conditions so that the oxygen would allow the mNeonGreen and mScarlet fluorophores to mature. Fluorescent signal above background was observed in all cells for both the *mNeonGreen* (Fig. 1A) and *mScarlet* constitutive reporters (Fig. 2A).

To quantify the fluorescent signal at the single-cell level, we adapted SuperSegger, a machine learning-based cell image analysis suite designed for time-lapse microscopy data (55), to calculate the mean fluorescent signal intensity for each cell in still images. SuperSegger readily identifies individual cells even if they are in close proximity, enabling automated quantification of thousands of individual cells. Using SuperSegger, we determined that the fluorescent signal was brighter for both the P*slpA*::*mNG* and P*slpA*::*mSc* reporters than for the analogous P*cwp2* reporters (Fig. 1B and 2B). Since the chromosomally encoded mNeonGreen reporters were bright enough to visualize above C. difficile’s autofluorescence, *mNeonGreen* and *mScarlet* could be suitable for use in chromosomally encoded dual-transcriptional-reporter systems. Importantly, chromosomal expression helps avoid issues related to variable plasmid copy number and plasmid segregation when examining the activity of gene-specific promoters at the single-cell level (39).

### mScarlet and mNeonGreen have distinct benefits and drawbacks for use in C. difficile.

To better understand the utility of the *mScarlet* and *mNeonGreen* reporters for such applications, we explored the advantages and disadvantages associated with the two reporters. This analysis was prompted by our finding that two populations of P*slpA*::*mNG* cells were often visible when their fluorescence intensities were plotted as a histogram (<12,000 relative fluorescence units [RFU]) (see Fig. S1 in the supplemental material), whereas P*cwp2*::*mNG* and the *mSc* constitutive reporter strains exhibited a largely normal distribution (Fig. S1). We hypothesized that P*slpA*::*mNG* cells with lower signal intensities (Fig. 1A, blue arrows; Fig. 1B) may experience toxicity related to high levels of mNeonGreen. Since a recent report showed that C. difficile cells expressing high levels of the *mCherry* reporter have reduced viability (39), we compared the growth of the *mNG* and *mSc* reporter strains in TY media relative to the wild type (WT) (no reporter). The optical density of the P*slpA*::*mNG* strain, but not the other strains (Fig. 1C), decreased after ~15 h of growth. Given that the P*slpA*::*mNG* strain produces considerably more mNeonGreen based on fluorescence measurements than the P*cwp2*::*mNG* strain (Fig. 1B), these data suggest that high levels of mNeonGreen protein are deleterious to the cell during late stationary phase.

10.1128/msphere.00132-22.1 FIG S1 High levels of mNeonGreen, but not mScarlet, can lead to a bimodal distribution. Frequency distribution analyses of P*slpA*::*mNG* reveals a bimodal distribution compared to the normal distributions observed with P*cwp2*::*mNG*, P*slpA*::*mSc*, and P*cwp2*::*mSc*. Values are mean fluorescence intensities of single cells grown overnight in TY broth. The first biological replicate is shown on the left and the second biological replicate is shown on the right for each graph. Download FIG S1, TIF file, 1.3 MB. Copyright © 2022 Donnelly et al. 2022 Donnelly et al. https://creativecommons.org/licenses/by/4.0/ This content is distributed under the terms of the Creative Commons Attribution 4.0 International license.

Since recent work has shown that C. difficile is more susceptible to autolysis during growth in brain heart infusion supplemented (BHIS) medium than TY media (56), we analyzed the behavior of our constitutive *mNG* and *mSc* reporter strains after culturing them overnight in BHIS broth. In contrast with the relatively uniform fluorescence of the reporter strains during overnight growth in TY broth (Fig. 1A), only a small fraction of P*slpA*::*mNG* and P*cwp2*::*mNG* cells were fluorescent following overnight growth in BHIS broth (Fig. S2A and B). This result suggests that either mNeonGreen is less stable in late-stationary-phase cells following growth in this medium or cells producing mNeonGreen lose their viability by this time point. Consistent with the latter hypothesis, the P*slpA*::*mNG* strain exhibited an even more severe decrease in optical density than the other strains during late-stationary-phase growth in BHIS broth (Fig. S2C). This issue was specific to *mNG*, because the P*slpA*::*mSc* and P*cwp2*::*mSc* strains retained their fluorescence even in overnight cultures in BHIS medium (Fig. S2B). A similar loss in mNeonGreen signal was observed with the P*slpA*::*mNG* reporter when strains were grown in BHIS medium regardless of whether the cells were fixed or live or whether they were grown on solid agar or in broth culture (data not shown). Taken together, these data suggest that high levels of mNeonGreen may be deleterious to C. difficile (56).

10.1128/msphere.00132-22.2 FIG S2 High levels of mNeonGreen can lead to toxicity in stationary-phase cultures. (A) Fluorescence microscopy analyses of fixed cells for wild type 630Δ*erm* (no reporter) and 630Δ*erm* carrying *mNeonGreen* coupled to either the *slpA* or *cwp2* constitutive promoters after overnight growth in BHIS broth (note that live-dead stain is not compatible with reporter system). Phase-contrast microscopy was used to visualize all bacterial cells. The merge of phase-contrast images and images with mNeonGreen pseudocolored in yellow is shown. (B) Fluorescence microscopy analyses of fixed cells for wild-type 630Δ*erm* (no reporter) and 630Δ*erm* carrying *mScarlet* coupled to either the *slpA* or *cwp2* constitutive promoter after overnight growth in BHIS broth. Phase-contrast microscopy was used to visualize all bacterial cells. The merge of phase-contrast and mScarlet pseudocolored in magenta is shown. (C) OD_600_ growth curve of the indicated strains during growth in BHIS broth. The graph is for a single biological replicate that is representative of three biological replicates. Download FIG S2, JPG file, 0.9 MB. Copyright © 2022 Donnelly et al. 2022 Donnelly et al. https://creativecommons.org/licenses/by/4.0/ This content is distributed under the terms of the Creative Commons Attribution 4.0 International license.

While high-level mScarlet production did not appear to strongly affect the growth of C. difficile, we noticed that the fluorescence of mScarlet-producing strains increased over time following exposure to oxygen relative to mNeonGreen-producing strains. This finding is consistent with mScarlet’s long maturation time of 174 min at 37°C (47) relative to mNeonGreen’s maturation time of 10 min at 37°C (45). To analyze the kinetics of mScarlet’s maturation in C. difficile cells upon exposure to oxygen, we measured its bulk fluorescent signal over time after exposing broth cultures of mScarlet-producing strains to ambient oxygen using a plate reader (Fig. 2C). The mScarlet signal started to peak around 6 h after fixation and exposure to oxygen, highlighting the importance of extended oxygen exposure for visualizing mScarlet. While mScarlet fluorescence was detectable in live cells even 1 h after exposure to oxygen, we found that waiting 2 h markedly increased the fluorescence level. Since oxygen exposures of >2 h resulted in chromosome fragmentation, we fixed samples containing the mScarlet fluorescent reporter with paraformaldehyde using previously established procedures (43). Notably, no difference in mNeonGreen fluorescence was observed between live and fixed cells, consistent with mNeonGreen’s short maturation time (45).

Despite mScarlet requiring fixation for maximal detection in C. difficile, we found that mScarlet is more sensitive than mNeonGreen, because C. difficile has minimal autofluorescence in the red channel, and mScarlet is less toxic in late stationary phase. Conversely, mNeonGreen is better suited for imaging live cells (i.e., without fixation) due to its short maturation time, although it can cause toxicity to C. difficile under certain growth conditions when produced in large amounts (Fig. 1C; Fig. S1C).

### Constitutive reporter fluorescence is reduced in sporulating cells especially in the forespore, but forespore-specific reporters can be generated.

**FIG 3:**
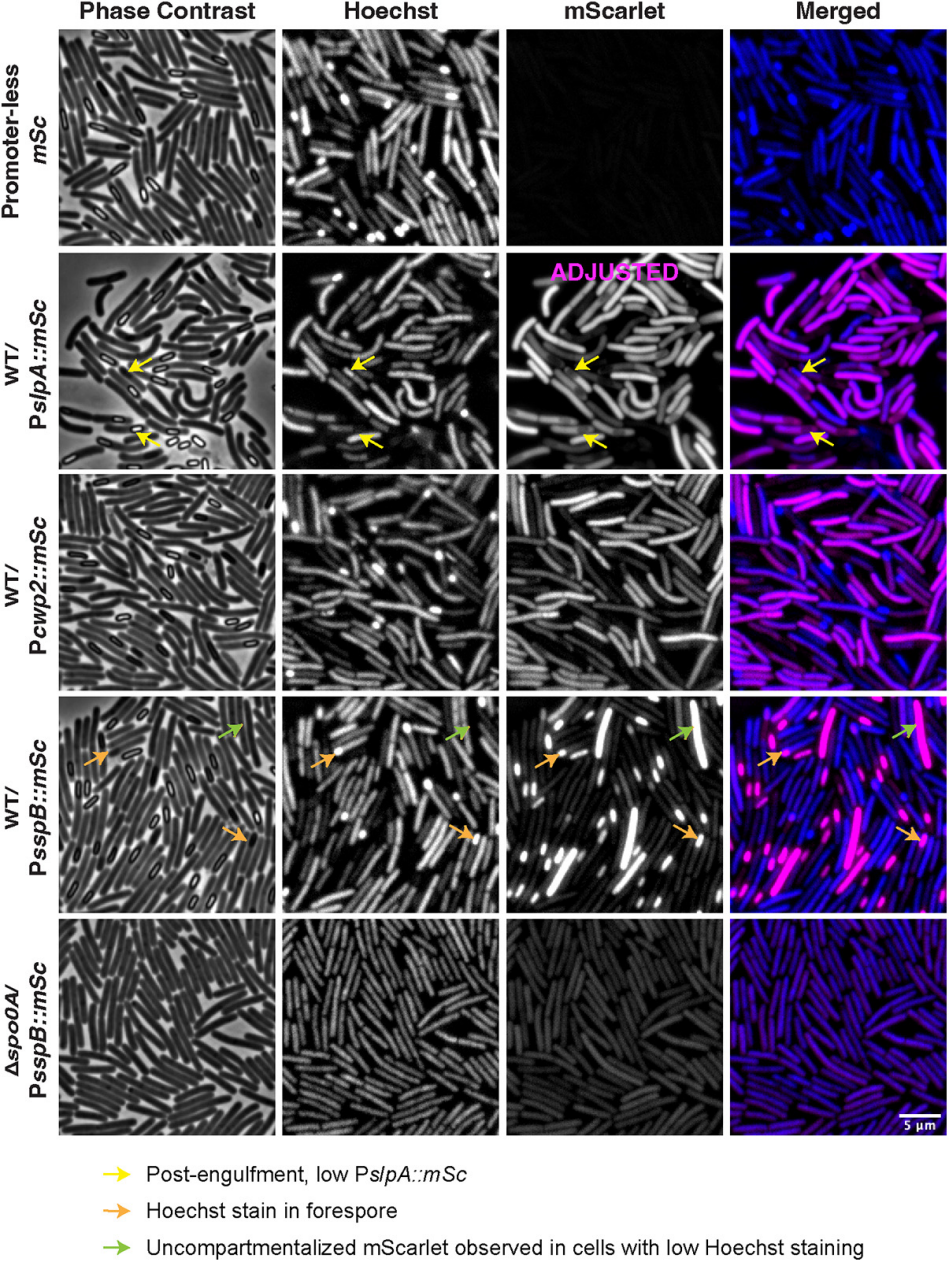
Expression levels of constitutive genes decrease in sporulating cells, particularly in the forespore, but forespore-specific gene expression can still be visualized. (A) Fluorescence microscopy analyses of P*slpA*::*mSc*, P*cwp2*::*mSc*, and P*sspB*::*mSc* reporter strains. Phase-contrast microscopy was used to visualize sporulating cells. Hoechst staining was used to visualize the nucleoid. The merge of the Hoechst staining (blue) and the mScarlet signal (magenta) is shown. Reduced mScarlet signal in the forespore of cells that are visibly sporulating is highlighted with a yellow arrow in the P*slpA*::*mSc* strain, but this was also observed for the P*cwp2*::*mSc* strain. Cells that are visibly sporulating based on the concentration of the Hoechst stain in the forespore are shown with orange arrows. Bright P*sspB*::*mScarlet* signal in cells that are either predivisional or sporulating cells where the forespore has become compromised and mScarlet has become distributed across the entire cell length in a Spo0A-dependent manner are highlighted by green arrows. Images are representative of three biological replicates. Microscopy was performed on live sporulating cultures 15 to 18 h after plating on SMC sporulation agar. The P*slpA*::*mSc* strain is displayed with adjusted settings to accommodate its bright signal relative to P*cwp2* and P*sspB* strains.

While analyzing constitutive reporter strains in different growth media, we observed that the fluorescence of the reporters appeared to decrease in the forespore region of cells undergoing sporulation (Fig. 1, pink arrows, and Fig. 3, yellow arrows). The decreased expression of genes encoding surface proteins like SlpA and Cwp2 in the forespore might be expected, because the mature spore does not produce these surface proteins (57). However, it is possible that the reduced signal for the constitutive reporters is an artifact caused by the inherent instability of the fluorescent proteins in the forespore. To address this possibility, we constructed *mNG* and *mSc* reporters driven by the forespore-specific *sspB* promoter (P*sspB*::*mNG* and P*sspB*::*mSc*) (19, 49). *sspB* encodes a small acid-soluble spore protein (SASP) that helps protect spore DNA from UV damage (58) and whose expression is σ^F-^ and σ^G-^ dependent and thus localized to the forespore (19, 49). Fluorescence in the P*sspB*::*mSc* reporter strain was localized to the forespore, indicating that it is possible for mScarlet to be stably produced in the forespore (Fig. 3). Surprisingly, the mScarlet signal was also occasionally observed throughout the cytosol of rod-shaped cells with no visible signs of sporulation based on their morphology (i.e., following asymmetric division [Fig. 3, green arrows]) (59). Since this apparent vegetative cell signal was not observed when we integrated the reporter into a Δ*spo0A* mutant (Fig. 3), which cannot initiate sporulation (25), the fluorescent signal could originate in cells that have induced sporulation but have not completed asymmetric division (i.e., predivisional cells) (60). In Bacillus subtilis, σ^F^ is aberrantly activated in WT predivisional cells with low frequency (0.5%) (Fig. 3) (60), but the frequency of uncompartmentalized mScarlet signal is ~10% in WT C. difficile cells. It is also possible that the mScarlet signal may have leaked out of the forespore, since this has been observed in some B. subtilis and C. difficile mutants with forespore membranes that are compromised by defects in assembly of the SpoIIQ-SpoIIIAH channel (61, 62). Unfortunately, in both these instances where uncompartmentalized sporulation reporter signal was observed in the literature, no nucleoid staining was performed. Accordingly, it is unclear why the cells with uncompartmentalized signal in Fig. 3 and Fig. S3 did not stain with Hoechst (Fig. 3, green arrows). Regardless of the origins of this signal, we note that a similar uncompartmentalized signal was observed for additional forespore-specific *mScarlet* and *mCherry* reporters that we tested involving fusions to the *spoIIQ*, *pdaA*, and *sspA* promoters (data not shown).

10.1128/msphere.00132-22.3 FIG S3 Expression levels of constitutive genes decrease in sporulating cells particularly in the forespore, but forespore-specific gene expression can still be visualized. (A) Fluorescent microscopy analyses of P*slpA*::*mNG*, P*cwp2*::*mNG*, and P*sspB*::*mNG* reporter strains. Phase-contrast microscopy was used to visualize sporulating cells (phase). Hoechst staining was used to visualize the nucleoid. The merge of the Hoechst (blue) and the mNeonGreen (yellow) signals is shown. Reduced mNeonGreen signal in the forespores of cells that are visibly sporulating is highlighted with a pink arrow in the P*slpA*::*mNG* strain, but this was also observed for the P*cwp2*::*mNG* strain. Cells that are visibly sporulating based on the concentration of the Hoechst stain in the forespore are shown with orange arrows. Images are representative of three biological replicates. Microscopy was performed on live sporulating cultures 15 to 18 h after plating on SMC sporulation agar. P*slpA*::*mNG* and P*cwp2*::*mNG* are displayed with adjusted settings to accommodate their bright signal relative to P*sspB*. Download FIG S3, JPG file, 1.1 MB. Copyright © 2022 Donnelly et al. 2022 Donnelly et al. https://creativecommons.org/licenses/by/4.0/ This content is distributed under the terms of the Creative Commons Attribution 4.0 International license.

While we readily detected the mScarlet signal in the forespore of sporulating P*sspB*::*mSc* cells, when we coupled the *sspB* promoter to *mNG*, mNeonGreen fluorescence in the forespore was barely above background autofluorescence (Fig. S3, orange arrows), highlighting how C. difficile's intrinsic autofluorescence reduces the sensitivity of *mNeonGreen*-based transcriptional reporters. Similar to the P*sspB*::*mScarlet* reporter, we also observed a “vegetative-cell-like” signal (Fig. S3, green arrows), indicating that it is not specific to mScarlet. Regardless, these experiments demonstrate that mNeonGreen and mScarlet can still be used to detect transcription in the forespore, implying that the reduced signal for P*slpA-* and P*cwp2-*driven reporters in the forespore and mother cell of sporulating cells likely reflects a general downregulation in gene expression as the forespore matures and the mother cell prepares to lyse and release the spore.

### Toxin gene expression appears elevated in strains with decreased sporulation.

Having established the utility of *mNeonGreen* and *mScarlet* transcriptional reporters for visualizing gene expression when chromosomally expressed in C. difficile, we next sought to investigate the relationship between toxin and sporulation gene expression at the single-cell level. We first generated chromosomal *mNG* and *mSc* reporters coupled to the *tcdA* promoter (P*tcdA*::*mNG* and P*tcdA*::*mSc*, respectively) to visualize the subpopulation of cells that express toxin genes. These constructs use the same promoter sequence as the plasmid-based P*tcdA*::*mCherry* transcriptional reporter previously described (18). As a control, we integrated P*tcdA*::*mNG* and P*tcdA*::*mSc* constructs into the *pyrE* locus of a clean *tcdR* deletion strain, because the TcdR alternative sigma factor activates *tcdA* expression (18). We also assessed how sporulation impacts toxin expression at the single-cell level by integrating the P*tcdA*::*mNG* and P*tcdA*::*mSc* reporters into the *pyrE* locus of a previously constructed Δ*spo0A* mutant, because prior work had suggested that Spo0A negatively regulates toxin production in some C. difficile strains (37) while other work suggests the opposite (63). To further explore the relationship between sporulation and toxin expression, we also moved the P*tcdA* reporter into a strain lacking the bifunctional transcriptional regulator RstA, which represses toxin production and promotes sporulation (34). While previous work had shown that *ΔrstA* overexpresses toxin genes in bulk population measurements, it was unclear whether loss of RstA impacts the frequency of cells expressing toxin genes and/or the magnitude of their toxin gene expression (34).

We validated our *ΔtcdR* and Δ*rstA* strains by complementing the respective mutants (no reporter) with a wild-type copy of *tcdR* or *rstA* driven by their native promoter expressed from the *pyrE* locus. Importantly, the Δ*tcdR*/*tcdR* complementation strain produced wild-type levels of TcdA in Western blot analyses of overnight TY broth cultures, while no TcdA was detected in the parental Δ*tcdR* strain (Fig. S4A). As expected, the Δ*rstA* mutant overproduced TcdA (~4.5-fold more; *P* < 0.0001), while the complementation strain produced wild-type levels of TcdA. Toxin levels were also elevated (~4-fold; *P* < 0.001) in the Δ*spo0A* mutant, while the complementation strain produced wild-type levels of TcdA.

10.1128/msphere.00132-22.4 FIG S4 Complementation of Δ*tcdR, Δspo0A*, and Δ*rstA* mutants. (A) Western blot analyses of TcdA from cell lysates prepared from TY medium overnight cultures. GDH was used as a loading control. Results for one replicate representative of three biological replicates is shown. (B) TcdA signal was quantified and normalized to the GDH loading control using the normalization by sum method (1). Dots represent the normalized values obtained from three biological replicates. (C) Ethanol resistance assay for measuring functional spore formation in the indicated strains after growth for 24 h on 70:30 medium. The Δ*spo0A* strain was used as a negative control. Δ*tcdR* and Δ*rstA* complemented strains appear comparable to the wild type, and the Δ*spo0A* complemented strain complements to levels greatly increased in comparison to the WT. Ethanol resistance values were normalized to wild-type values; representative phase-contrast images of sporulating cultures are displayed. Download FIG S4, JPG file, 0.5 MB. Copyright © 2022 Donnelly et al. 2022 Donnelly et al. https://creativecommons.org/licenses/by/4.0/ This content is distributed under the terms of the Creative Commons Attribution 4.0 International license.

**FIG 4:**
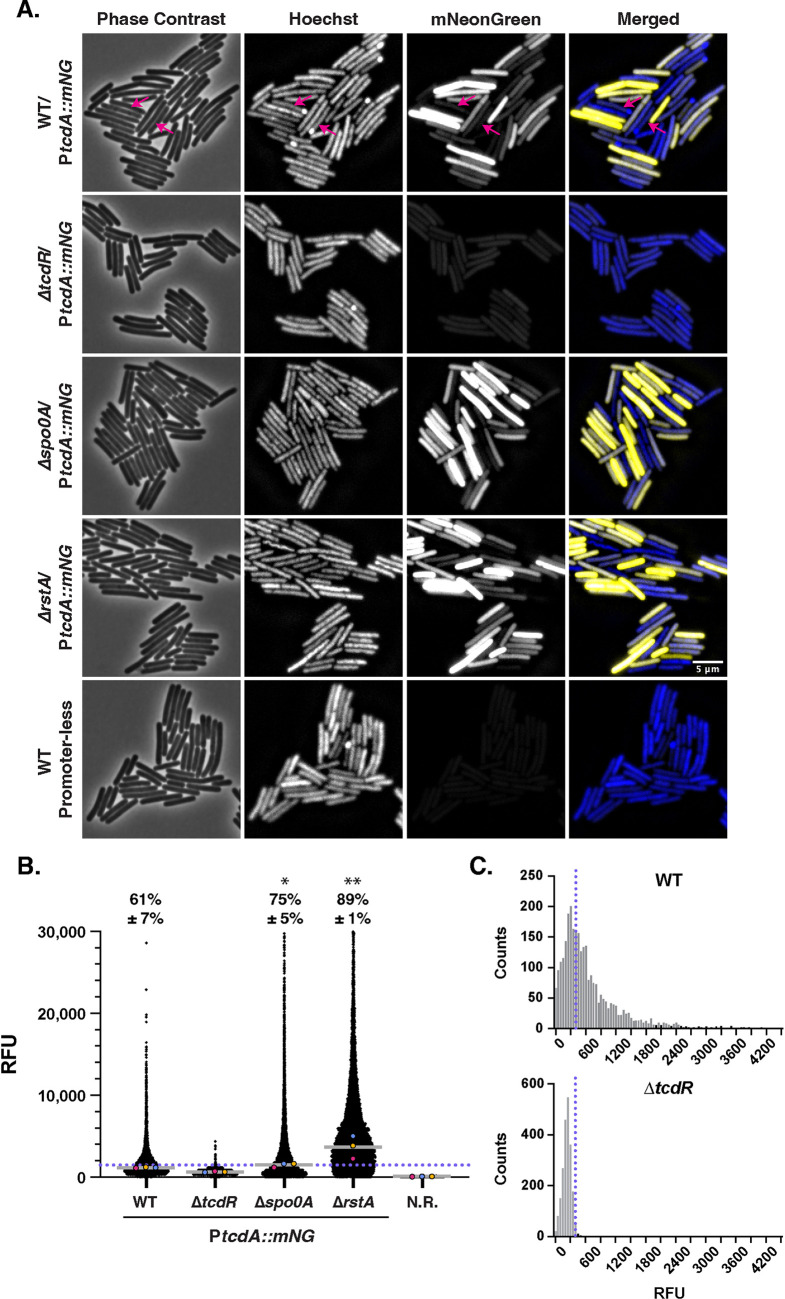
Toxin gene expression is elevated in strains with reduced sporulation (for both mNG and mSc reporters). (A) Fluorescence microscopy analyses of live cells from strains carrying P*tcdA*::*mNG* toxin gene reporters grown overnight in TY broth relative to a promoterless *mNG* construct integrated into the *pyrE* locus. Phase-contrast microscopy was used to visualize all bacterial cells, and the nucleoid was stained with Hoechst. The merge of images showing Hoechst staining (blue) and mNeonGreen pseudocolored in yellow is shown. Sporulating cells, detected based on Hoechst stain with decreased toxin reporter levels, are highlighted with magenta arrows. (B) SuperSegger-based quantification of the mean fluorescence intensity of each cell is shown as a black dot on the scatterplot. Individual cell intensities were quantified from three biological replicates with at least two fields of view per strain per replicate. The magenta, yellow, and blue dots represent the median intensities for the first, second, and third biological replicates, respectively. The gray line represents the mean value of each replicate’s median value. N.R., strain harboring *mNeonGreen* with no upstream promoter region integrated into the *pyrE* locus. The percent Toxin-ON is displayed, with the standard deviation. Toxin-ON cells were calculated using the 99th percentile of the Δ*tcdR* signal as a cutoff (value displayed as a blue dotted line). A minority of points (<1%) are outside the limits of the scatterplot; axes were adjusted to provide an optimal view of the scatterplot trends. Statistical significance was determined relative to wild type using a one-way ANOVA and Tukey’s test. *, *P* < 0.1; **, *P* < 0.01. (C) Histogram analysis of single-cell fluorescence intensities for the P*tcdA*::*mNG* reporter in the wild type versus the *ΔtcdR* strain. The 99th percentile cutoff in the Δ*tcdR* strain used to define cells as Toxin-ON is shown by the blue dotted line. Plotted data are a compilation of data from three biological replicates.

When P*tcdA*::*mNG* and P*tcdA*::*mSc* were integrated into the *pyrE* locus of wild-type 630Δ*erm*, the reporters exhibited a heterogeneous expression pattern within the population of cells (Fig. 4A; Fig. S5) similar to previously described analyses using a plasmid-based reporter (18). Some cells were clearly in a “Toxin-ON” state, with bright mNeonGreen or mScarlet fluorescence visible, and other cells lacked detectable fluorescence and were thus “Toxin-OFF” (18). In the strain 630 background, this heterogeneity is a function of variation in *tcdR* expression levels, since inducible expression of *tcdR* breaks the positive feedback loop that controls *tcdR* expression and thus bistable expression of the *tcdA* gene (18). As expected, little signal was observed in the Δ*tcdR* mutant background. We also attempted to visualize *tcdB* toxin gene expression using *mNeonGreen* and *mScarlet* reporters in C. difficile, but both chromosomal and plasmid-based reporters failed to generate fluorescent signal above background (data not shown).

10.1128/msphere.00132-22.5 FIG S5 Toxin gene expression is elevated in strains with reduced sporulation (mScarlet reporter). (A) Fluorescence microscopy analyses of live cells from strains carrying P*tcdA*::*mSc* toxin gene reporters grown overnight in TY broth relative to a promoterless *mSc* construct integrated into the *pyrE* locus. Phase-contrast microscopy was used to visualize all bacterial cells, and the nucleoid was stained with Hoechst. The merge of Hoechst (blue) and mScarlet (magenta) images is shown. Sporulating cells based on Hoechst stain with decreased toxin reporter levels are highlighted with yellow arrows. (B) SuperSegger-based quantification of the mean fluorescence intensity of each cell is shown as a black dot on the scatterplot. Individual cell intensities were quantified from three biological replicates with at least two fields of view per strain per replicate. The magenta, yellow, and blue dots represent the median intensities for the first, second, and third biological replicates, respectively. The gray line represents the mean value of each replicate’s median value. N.R., strain harboring *mNeonGreen* with no upstream promoter region integrated into the *pyrE* locus. The percent Toxin-ON is displayed, with the standard deviation. Toxin-ON cells were calculated using the 99th percentile of the Δ*tcdR* signal as a cutoff (value displayed as a blue dotted line). A minority of points (<1%) are outside the limits of the scatterplot; axes were adjusted to provide an optimal view of the scatterplot trends. Statistical significance was determined relative to wild-type values using a one-way ANOVA and Tukey’s test. *, *P* < 0.1; ** *P* < 0.01. (C) Histogram analysis of single-cell fluorescence intensities for the P*tcdA*::*mSc* reporter in wild-type versus *ΔtcdR* cells. The 99th percentile cutoff in Δ*tcdR* cells used to define cells as Toxin-ON is shown by the blue dotted line. Plotted data are a compilation of data from three biological replicates. Download FIG S5, JPG file, 1.3 MB. Copyright © 2022 Donnelly et al. 2022 Donnelly et al. https://creativecommons.org/licenses/by/4.0/ This content is distributed under the terms of the Creative Commons Attribution 4.0 International license.

To quantify how many cells were in the Toxin-ON state with our P*tcdA* reporters, we set the cutoff for wild-type P*tcdA* expression as above the 99th percentile of the P*tcdA* reporters in the Δ*tcdR* strain background. With this cutoff, 61% of cells in the WT population were Toxin-ON in the *mNeonGreen* reporter strain (Fig. 4B) and 37% were Toxin-ON in the *mScarlet* reporter strain cells (Fig. S5 and S6). While the fraction of Toxin-ON cells appears to be different between the P*tcdA*::*mNG* and P*tcdA*::*mSc* reporter strains, we note that slight changes in the cutoff value for Toxin-ON cells result in a 5 to 10% change in the proportion of Toxin-ON cells. Extending the maturation time beyond the 2 h at 37°C that we used might also have increased the proportion of Toxin-ON cells detected with the P*tcdA*::*mScarlet* reporter by increasing mScarlet fluorescence above the Δ*tcdR* background. While the biological significance of a cell that is just under or over the cutoff value set by Δ*tcdR* remains unclear, our single-cell analyses confirm that there is a broad range in toxin reporter expression within wild-type cells.

10.1128/msphere.00132-22.6 FIG S6 Triplicate analyses of toxin gene reporters in TY broth. Scatterplots of triplicate measurements of P*tcdA*::*mSc* strains (A) versus P*tcdA*::*mNG* strains (B) using SuperSegger to assess reproducibility across the three biological replicates. The mean fluorescence intensity of each cell is displayed as a black dot on the scatterplot. The colored line represents the median intensity for each strain. The blue dotted line represents (A) the 99th percentile of the Δ*tcdR* signal as a cutoff or (B) the sporulation cutoff determined by cytological analyses (1,000 RFU). (C) Histogram analyses of single-cell fluorescence intensities for (A) P*tcdA*::*mSc* and (B) P*tcdA*::*mNG* demonstrate a long-tailed distribution. Values displayed represent the mean fluorescence intensities of single cells grown overnight in TY broth. The 99th percentile cutoff in Δ*tcdR* used to define cells as Toxin-ON is shown by the blue dotted line. Δ*spo0A* and Δ*rstA* cells demonstrate a similar long-tailed distribution, with increased toxin expression compared to the wild type. Data plotted are a compilation of data from three biological replicates. Download FIG S6, PDF file, 0.5 MB. Copyright © 2022 Donnelly et al. 2022 Donnelly et al. https://creativecommons.org/licenses/by/4.0/ This content is distributed under the terms of the Creative Commons Attribution 4.0 International license.

Our findings with these chromosomal toxin reporters are similar to those of prior analyses using a plasmid-based P*tcdA*::*mCherry* reporter, which found that ~80% of cells are Toxin-ON in the 630 strain background (18). The slight differences in Toxin-ON frequencies between our studies could be due to plasmid copy number variation and/or the greater sensitivity of the plasmid-based reporter due to its multicopy nature. Regardless, our chromosomal P*tcdA* fluorescent reporters gave a distribution of Toxin-ON cells similar to that of a plasmid-based P*tcdA*::*mCherry* reporter (18) when plotted as a histogram (Fig. S6C). Notably, the P*tcdA*::*mNG* reporter exhibited a long-tailed distribution similar to that of the P*tcdA*::*mCherry* plasmid-based reporter in the 630 strain background, in contrast with the bimodal distribution in toxin gene expression observed for some C. difficile strains (18).

Notably, the long-tailed distribution in toxin reporter expression was even greater for Δ*spo0A* and Δ*rstA* cells (Fig. S6C). Δ*spo0A* cells exhibited an ~20% increase in the magnitude of toxin gene expression at the single-cell level and an ~10 to 15% increase in the frequency of Toxin-ON cells (75% for mNG and 48% for mSc reporters relative to WT [Fig. 4; Fig. S5]). Δ*rstA* cells expressed the toxin reporter at higher levels (~2-3-fold increase relative to WT), and almost every Δ*rstA* cell was Toxin-ON (89% for *mNG* and 78% for *mSc* cells). These results are largely consistent with the elevated TcdA levels observed in Δ*spo0A* and Δ*rstA* mutants (Fig. S4) and the previous observation that loss of RstA derepresses toxin gene expression in bulk (34, 35). Since the loss of either Spo0A or RstA increases the proportion of cells expressing the toxin reporters as well as the magnitude of their expression, and since the Δ*spo0A* strain cannot sporulate and the Δ*rstA* strain sporulates at reduced levels, our results suggest that toxin and sporulation genes might be inversely related.

### Sporulation is not elevated in a toxinless strain.

**FIG 5:**
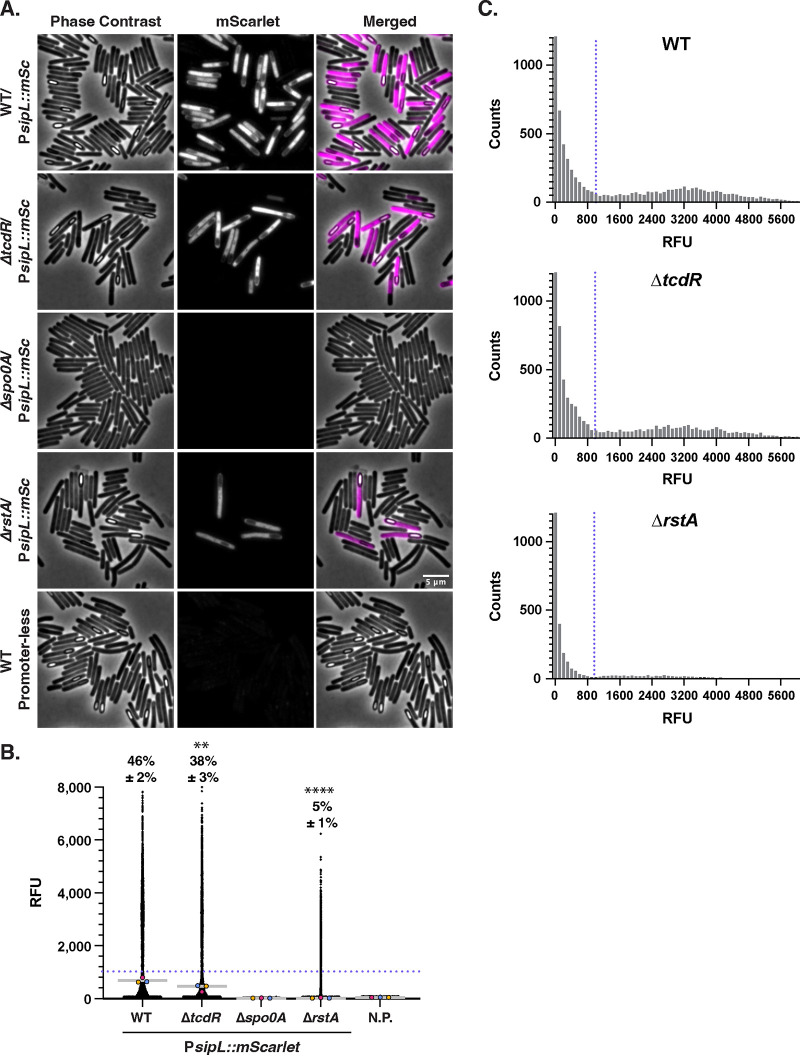
Sporulation gene expression is reduced in a toxin-less strain. (A) Fluorescence microscopy analyses of sporulating cultures of the indicated strains grown for 15 to 18 h on SMC sporulation agar followed by fixation. Phase-contrast microscopy was used to visualize all bacterial cells. The merge of Hoechst (blue) and mScarlet (magenta) images is shown. (B) SuperSegger-based quantification of the mean fluorescence intensity of each cell is shown as a black dot on the scatterplot. Individual cell intensities were quantified from three biological replicates, with at least two fields of view per strain per replicate. The magenta, yellow, and blue dots represent the median intensities for the first, second, and third biological replicates, respectively. The gray line represents the mean value of each replicate’s median value. N.R., strain harboring *mScarlet* with no upstream promoter region integrated into the *pyrE* locus. The percent Sporulation-ON is displayed, with the standard deviation, and was calculated using a signal of 1,000 RFU as a cutoff (value displayed as a blue dotted line). This cutoff was determined using histogram analyses (Fig. 5). A minority of points (<1%) are outside the limits of the scatterplot; axes were adjusted to provide an optimal view of the scatterplot trends. Statistical significance was determined relative to wild-type values using a one-way ANOVA and Tukey’s test. *, *P* < 0.1; ** *P* < 0.01. (C) Histogram analysis of mean single-cell fluorescence intensities for the P*sipL*::*mSc* reporter in wild-type, *ΔtcdR*, and Δ*rstA* strains demonstrates a bimodal distribution of cells as Sporulation-ON versus Sporulation-OFF. The blue dotted line indicates the determined cutoff value of 1,000 RFU, which was also confirmed by visual inspection of phase-contrast images. Plotted data are a compilation of data from three biological replicates.

Consistent with this hypothesis, sporulating cells in these samples (visible based on their Hoechst staining) were frequently observed to be Toxin-OFF (Fig. 4A, pink arrows). To assess whether toxin gene expression decreases sporulation, we visualized C. difficile sporulation gene expression in strains that either cannot produce toxin (Δ*tcdR*) or produce elevated levels of toxin (Δ*rstA*). To this end, we coupled the sporulation-specific *sipL* promoter to mNeonGreen and mScarlet (P*sipL*::*mNG* and P*sipL*::*mSc*, respectively). *sipL* is expressed under the control of the mother cell-specific sigma factor σ^E^ (19, 49), so its expression should localize only to the mother cell cytosol and not the forespore. We integrated the P*sipL*::*mNG* and P*sipL*::*mSc* sporulation reporters into the *pyrE* loci of WT, Δ*tcdR*, Δ*spo0A*, and Δ*rstA* strains and visualized their expression on sporulation media (SMC agar). As expected, the P*sipL*::*mNG* and P*sipL*::*mSc* fluorescence was observed only in the mother cell for all strains analyzed, and no signal above background was observed for Δ*spo0A* (19, 49) (Fig. 5; Fig. S7). While the signal for P*sipL*::*mSc* was much brighter than for P*sipL*::*mNG* (Fig. 5; Fig. S7A), the P*sipL*::*mNG* reporter was useful for detecting *sipL* gene expression in live, sporulating cells that had completed asymmetric division but not engulfment (Fig. S7A, pink arrows). Cells at this stage of sporulation stain brightly in the forespore with Hoechst because the nucleoid is concentrated in a small region (59). This morphological information is largely lost for the P*sipL*::*mSc* reporter, because the fixation procedure used to enhance the mScarlet signal causes the chromosome to fragment and obscures the forespore nucleoid signal (Fig. S5). In contrast, the P*sipL*::*mNG* reporter can be used with the membrane stain FM4-64 and the nucleoid stain Hoechst (Fig. S7B) to glean useful cytological information on a cell’s sporulation stage (59) as well as additional cellular phenotypes (64). Unfortunately, the mScarlet reporter is not compatible with the FM4-64 stain, limiting the utility of this reporter in cytological profiling studies.

10.1128/msphere.00132-22.7 FIG S7 Analyses of sporulation with the P*sipL*::*mNG* reporter. (A) Fluorescence microscopy analyses of live cell sporulating cultures of the indicated strains grown for 15 h on SMC sporulation agar. Phase-contrast microscopy was used to visualize all bacterial cells. The merge of Hoechst (blue) and mNeonGreen (yellow) images is shown. Signal was not sufficiently bright over autofluorescence to perform SuperSegger quantitative image analysis. Pink arrows indicate sporulating cells that have completed asymmetric division but not engulfment. (B) P*sipL*::*mNG* reporter strain on SMC plates is shown to be compatible with the FM4-64 membrane signal. Phase contrast shows all cells and spores. The merge of Hoechst (blue), mNeonGreen (yellow), and FM464 (magenta) images is shown. Green arrows indicate sporulating cells that have completed asymmetric division but have not yet initiated engulfment or activated σ^E^ and thus do not express the P*sipL*::*mScarlet* reporter. Download FIG S7, JPG file, 1.4 MB. Copyright © 2022 Donnelly et al. 2022 Donnelly et al. https://creativecommons.org/licenses/by/4.0/ This content is distributed under the terms of the Creative Commons Attribution 4.0 International license.

Despite these advantages, the P*sipL*::*mNG* reporter was too dim to be reliably quantified above C. difficile’s autofluorescence (data not shown). Quantification of the P*sipL*::*mSc* reporter allowed us to visualize two distinct populations of sporulating cells and nonsporulating cells (Fig. 5A and B). A similar distribution of sporulating cells (Fig. 5B, population to the right of the dashed line) was observed for the WT and Δ*tcdR* P*sipL*::*mSc* reporter strains, whereas far fewer cells were found to be sporulating in the Δ*rstA* reporter strain, consistent with the reduced sporulation phenotype of a Δ*rstA* strain (Fig. 5B). When we attempted to quantify the proportion of cells that were “Sporulation-ON” using the same method as the toxin reporter calculations, i.e., using the 99th percentile of the Δ*spo0A* signal as the cutoff, 100% of wild-type cells were determined to be sporulating. Since this was clearly not reflected in microscopy analyses (phase-contrast and visualizing the reporter signal), we defined the Sporulation-ON population based on histogram analyses (Fig. 5B) and manual inspection of the fluorescent signal in visibly sporulating cells versus nonsporulating cells. With this cutoff, 46% of wild-type and 38% of Δ*tcdR* cells were identified as Sporulation-ON (Fig. 5C). This slight decrease in proportion was statistically significant (*P* < 0.005); a slight reduction in the median fluorescence of the population was also observed between the Δ*tcdR* strain and the WT. Notably, the magnitude of the P*sipL*::*mSc* signal at the single-cell level decreased in the Δ*rstA* strain relative to the WT, and only 5% of cells were found to be Sporulation-ON (9-fold decrease; *P* < 0.0001).

The reporter data were largely consistent with our functional analyses of sporulation using an ethanol resistance assay performed on 70:30 plates as previously described (Fig. S4C). The Δ*tcdR* strain produced functional spores at wild-type levels, while the Δ*rstA* strain produced ~3-fold fewer spores. In general, we detected smaller differences in sporulation efficiency than in prior studies, where a *tcdR* Targetron insertion mutant made ~2-fold more spores than wild-type 630Δ*erm* (36) and an *rstA* Targetron insertion mutant made 7- to 23-fold fewer spores than wild-type 630Δ*erm* (34, 35). However, since the ethanol resistance assay measures both sporulation and germination efficiency, this assay tends to be more variable than the reporter assays (Fig. S8) in our hands. This difference in variability is reflected in the statistical significance of the results obtained for each assay, with the reduction in sporulation for Δ*tcdR* and Δ*rstA* mutants relative to wild type, achieving statistical significance for the reporter analyses (Fig. 5C) but not the ethanol resistance assays. Regardless, these analyses validate the utility of the P*sipL*::*mSc* reporter for detecting cells that have completed asymmetric division.

10.1128/msphere.00132-22.8 FIG S8 Triplicate analyses of the P*sipL*::*mSc* sporulation gene reporter on SMC plates. Scatterplots of triplicate measurements of *PsipL*::*mSc* strains to assess the reproducibility across the three biological replicates. The mean fluorescence intensity of each cell is displayed as a black dot on the scatterplot. The colored line represents the median intensity for each strain. The pink dotted line represents 1,000 RFU as a sporulation cutoff. Download FIG S8, TIF file, 0.4 MB. Copyright © 2022 Donnelly et al. 2022 Donnelly et al. https://creativecommons.org/licenses/by/4.0/ This content is distributed under the terms of the Creative Commons Attribution 4.0 International license.

### Certain growth conditions promote a division of labor between transmission and virulence gene expression.

Having validated the single reporters P*tcdA*::*mNG* and P*sipL*::*mSc*, we next constructed dual-reporter strains expressing both P*tcdA*::*mNG* and P*sipL*::*mSc* in the WT, Δ*tcdR*, Δ*spo0A*, and Δ*tcdR* strain backgrounds. These dual-reporter strains allow us to directly visualize at the single-cell level the extent to which sporulating subpopulations and toxin-expressing subpopulations overlap (or bifurcate) in a coordinated manner, or whether the *tcdA* and *sipL* genes are heterogeneously expressed independently of the other. The reporters also allow us to simultaneously assess the effect of media and growth conditions on toxin and sporulation gene expression. To construct the chromosomal dual-reporter strains, the P*sipL*::*mSc* reporter was first inserted downstream of the *sipL* locus using allelic exchange, and then the P*tcdA*::*mNG* reporter was integrated into the *pyrE* locus. The magnitude and proportion of cells expressing toxin and sporulation genes at the single-cell level were quantified using the cutoff values defined for the single reporters, since the toxin and sporulation gene reporters were expressed at similar levels and proportions for the WT dual-reporter strain and the single reporter strain.

**FIG 6:**
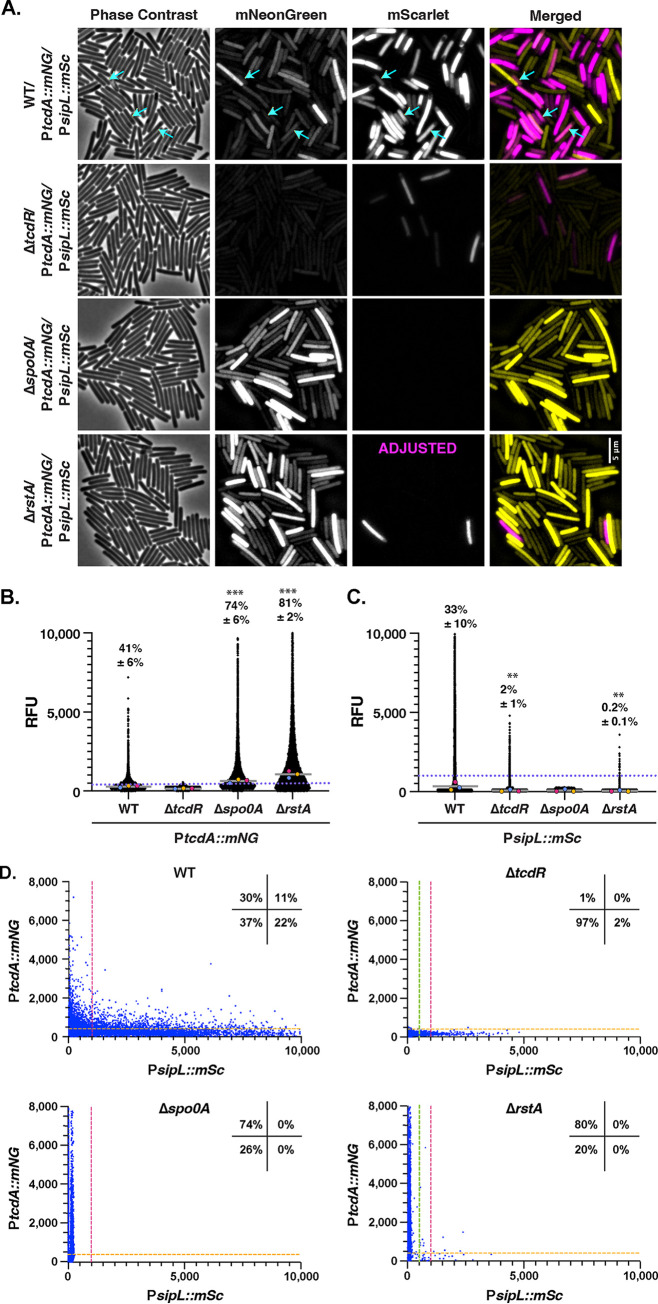
Simultaneous toxin and sporulation gene expression are observed in a subset of WT but not Δ*rstA* mutant cells during stationary-phase growth in TY broth. (A) Fluorescence microscopy analyses of fixed bacterial cells after overnight growth in TY liquid media. Dual-reporter strains contain P*sipL*::*mScarlet* and P*tcdA*::*mNeonGreen.* Dual-reporter analyses were visualized in WT, Δ*tcdR*, Δ*spo0A*, and Δ*rstA* strain backgrounds. Phase-contrast microscopy was used to visualize all bacterial cells. The merge of mNeonGreen (yellow) and mScarlet (magenta) signals is shown. Cells expressing both P*sipL*::*mScarlet* and P*tcdA*::*mNeonGreen* are highlighted with blue arrows. (B and C) SuperSegger-based quantification of P*tcdA*::*mNG* and P*sipL*::*mSc* reporters shows the mean fluorescence intensity of each cell as a black dot on the scatterplot for the indicated reporters. Individual cell intensities were quantified from three biological replicates, with at least two fields of view per strain per replicate. The magenta, yellow, and blue dots represent the median intensities for the first, second, and third biological replicates, respectively. The gray line represents the mean value of each replicate’s median value. The percent Toxin-ON is displayed, with the standard deviation. Toxin-ON cells were calculated using the 99th percentile of the Δ*tcdR* signal as a cutoff (value displayed as an orange dotted line). The percent Sporulation-ON was calculated using the 1,000-RFU signal as a cutoff (value displayed as a pink dotted line; the green dotted line represents the 500-RFU cutoff for Sporulation-ON cells for the Δ*tcdR* and Δ*spo0A* strain). This cutoff was determined using the histogram analyses in Fig. 5B. A minority of points (<1%) are outside the limits of the scatterplot; axes were adjusted to provide an optimal view of the scatterplot trends. Statistical significance was determined relative to wild-type values using a one-way ANOVA and Tukey’s test. ***, *P* < 0.001; ** *P* < 0.01.

When the P*tcdA*::*mNG*-P*sipL*::*mSc* dual-reporter strains were grown overnight in TY broth, which is traditionally used to induce maximal toxin gene expression (20), 30% ± 5% of the wild-type population was solely Toxin-ON and 21% ± 6% of the population was solely Sporulation-ON (Fig. 6D). Simultaneous expression of both *tcdA* and *sipL* was observed in 11% ± 3% of the population (Fig. 6, blue arrows) such that 41% ± 6% of the overall bacterial population was Toxin-ON (similar to what we observed in the single reporter strains in TY broth) (Fig. 4). About one-third of the population was Sporulation-ON, indicating that a sizable proportion of cells induce sporulation genes in TY broth, a property that was not possible to accurately quantify in our earlier analyses with the single P*tcdA*::*mNG* toxin reporter (Fig. 4). In TY broth, Δ*tcdR* induced the *sipL* reporter at low levels, with only 2% ± 1% of cells being identified as Sporulation-ON (Fig. 6C). However, when we used the bimodal distribution of the P*sipL*::*mSc* reporter from the Δ*tcdR* dual-reporter strain to set the cutoff for Sporulation-ON cells (500 RFU [(Fig S6C)]), approximately ~4% of Δ*tcdR* cells were identified as Sporulation-ON in TY broth cultures. Importantly, this proportion more accurately reflects the proportion of cells with visual signs of sporulation through inspection of the fluorescence microscopy images (Fig. 6).

Consistent with our analyses of the single reporter P*tcdA*::*mSc* in TY broth, the Δ*spo0A* and Δ*rstA* dual-reporter strains induced the toxin reporter in a high proportion of cells (74% ± 6% and 81% ± 2%, respectively) and to higher levels than the WT (Fig. 4 and 6). Interestingly, only a small fraction of Δ*rstA* cells were identified as Sporulation-ON (0.2%). Taken together, these analyses indicate that overnight TY broth culture strongly induces both toxin and sporulation gene expression in wild-type cells. This result implies that these two processes likely are independent of each other, since similar proportions of cells appear to express both toxin and sporulation genes. Conversely, in the Δ*rstA* strain, very little overlap between these processes was observed, with cells primarily expressing *tcdA* often to high levels and a small subset inducing *sipL*. This result suggests that RstA functions to reduce the “commitment” of the population to one process or the other, since the loss of RstA increases the frequency and magnitude of toxin gene expression and decreases the frequency and magnitude of sporulation gene expression relative to the WT.

**FIG 7:**
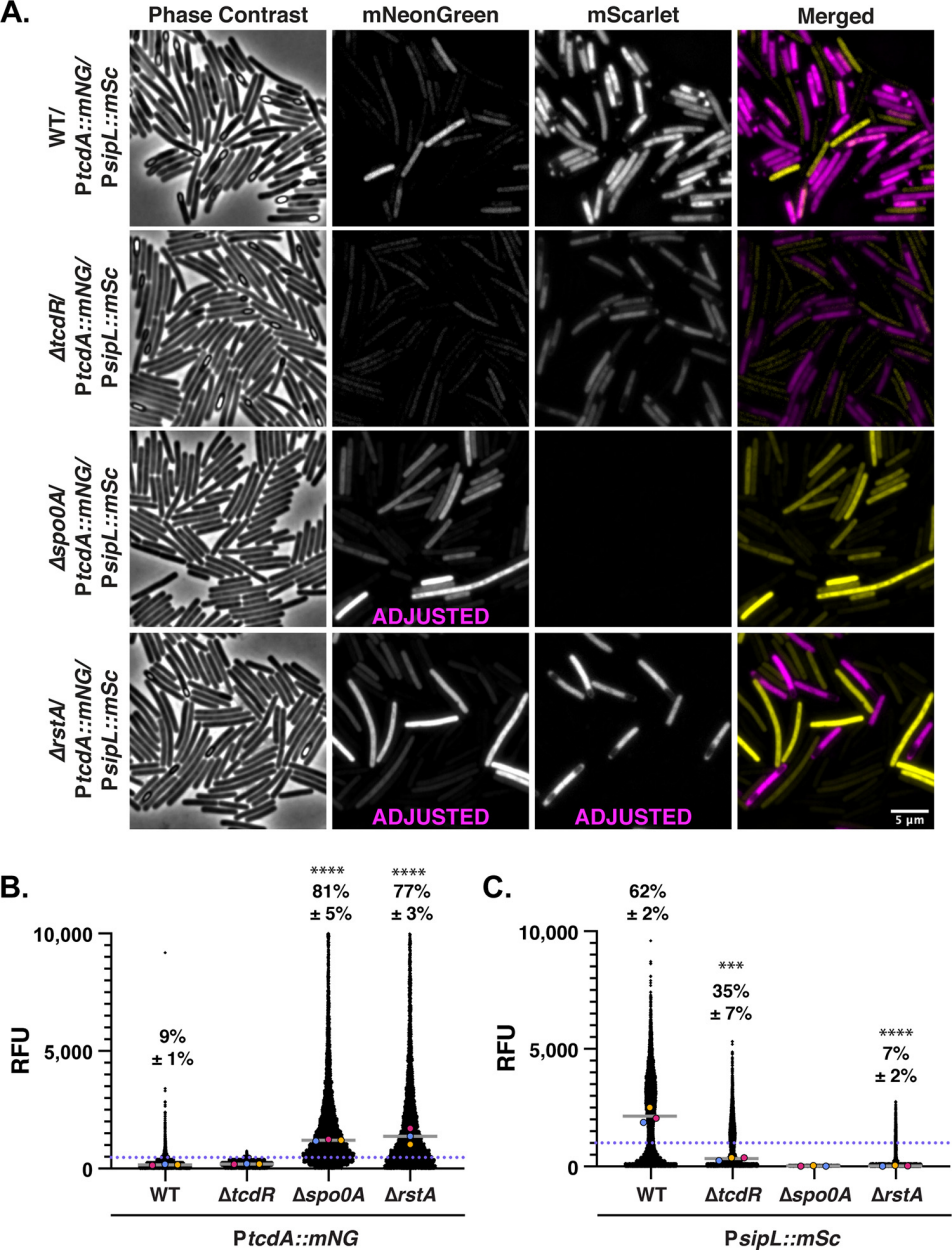

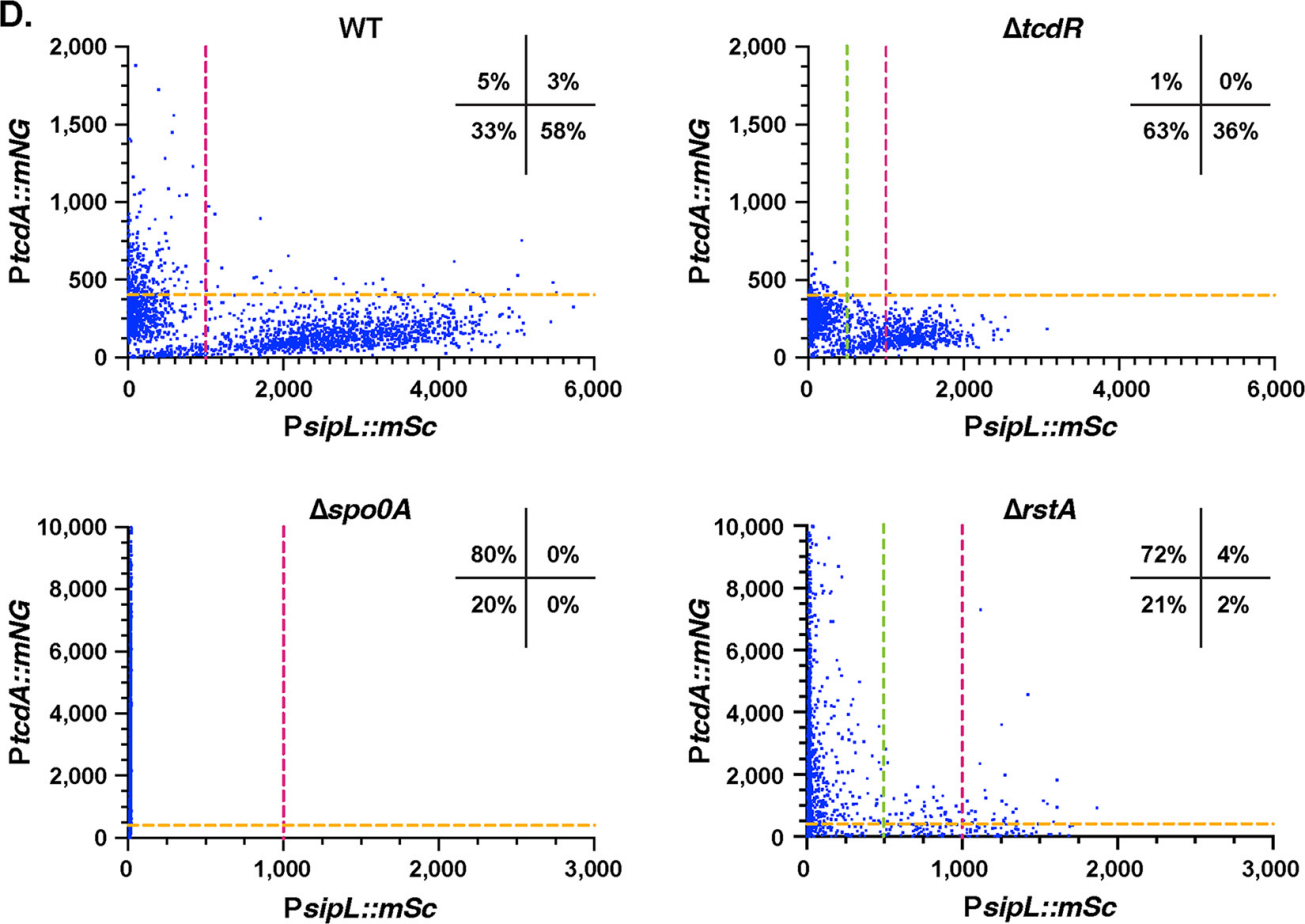
Minimal overlap between toxin and sporulation gene expression is observed during growth on TY plates. (A) Fluorescence microscopy analyses of fixed bacterial cells after growth on TY plates. Dual-reporter strains contain P*sipL*::*mScarlet* and P*tcdA*::*mNeonGreen.* Dual-reporter analyses were visualized in WT, Δ*tcdR*, Δ*spo0A*, and Δ*rstA* strain backgrounds. Phase-contrast microscopy was used to visualize all bacterial cells. The merge of mNeonGreen (yellow) with mScarlet (magenta) signals is shown. (B and C) SuperSegger-based quantification of P*tcdA*::*mNG* and P*sipL*::*mSc* reporters shows the mean fluorescence intensity of each cell as a black dot on the scatterplot for the indicated reporters. Individual cell intensities were quantified from three biological replicates, with at least two fields of view per strain per replicate. The magenta, yellow, and blue dots represent the median intensities for the first, second, and third biological replicates, respectively. The gray line represents the mean value of each replicate’s median value. The precent Toxin-ON is displayed, with the standard deviation. Toxin-ON cells were calculated using the 99th percentile of the Δ*tcdR* signal as a cutoff (value displayed as a blue dotted line). The percent Sporulation-ON was calculated using the 1,000-RFU signal as a cutoff (value displayed as a pink dotted line; the green dotted line represents the 500-RFU cutoff for Sporulation-ON cells for Δ*tcdR* and Δ*spo0A* strains). This cutoff was determined using the histogram analyses in Fig. 5B A minority of points (<1%) are outside the limits of the scatterplot; axes were adjusted to provide an optimal view of the scatterplot trends. Statistical significance was determined relative to wild type using a one-way ANOVA and Tukey’s test. ***, *P* < 0.001; ** *P* < 0.01. (D) Scatterplot analyses show single-cell mean fluorescence intensities, with P*sipL*::*mSc* on the *x* axis and *PtcdA*::*mNG* on the *y* axis. The Toxin-ON cutoff is represented by the orange dotted line, which indicates the 99th percentile of the Δ*tcdR* signal. The Sporulation-ON cutoff is represented by the pink dotted line at 1,000 RFU based on the histogram bimodal distribution analyses; the green line highlights the Sporulation-ON cutoff for Δ*tcdR* and Δ*rstA* based on the histogram analyses. Percentages indicate Toxin-ON/Sporulation-OFF in the top left quadrant, Toxin-ON/Sporulation-ON in the top right quadrant, Toxin-OFF/Sporulation-OFF in the bottom left quadrant, and Toxin-OFF/Sporulation-ON in the bottom right quadrant. A minority of points (<1%) are outside the limits of the scatterplot; axes were adjusted to provide an optimal view of the scatterplot trends.

Since toxin and sporulation gene expression are highly responsive to environmental conditions, we tested how different growth conditions affected their expression at the single-cell level. As a comparison to the TY liquid medium mentioned above, we analyzed the behavior of the dual-reporter strains on TY plates (Fig. 7). While TY broth strongly induces toxin gene expression in the WT (20), when the WT was grown on TY agar, it was 5-fold less likely to induce toxin (9% versus 41% of the population was Toxin-ON on TY agar versus TY broth), and the magnitude of toxin gene expression was also ~2-fold lower on TY agar versus TY broth (Fig. 6 and 7). In contrast, growth of WT on TY plates increased the frequency of sporulation by ~2-fold (62% versus 33% of cells were Sporulation-ON on TY agar versus TY broth). Notably, when toxin gene expression was plotted against sporulation gene expression, minimal overlap was observed between the two populations for WT, with only 2% ± 0.1% simultaneously expressing toxin and sporulation genes when grown on TY plates, compared to 11% ± 3% in TY broth.

**FIG 8:**
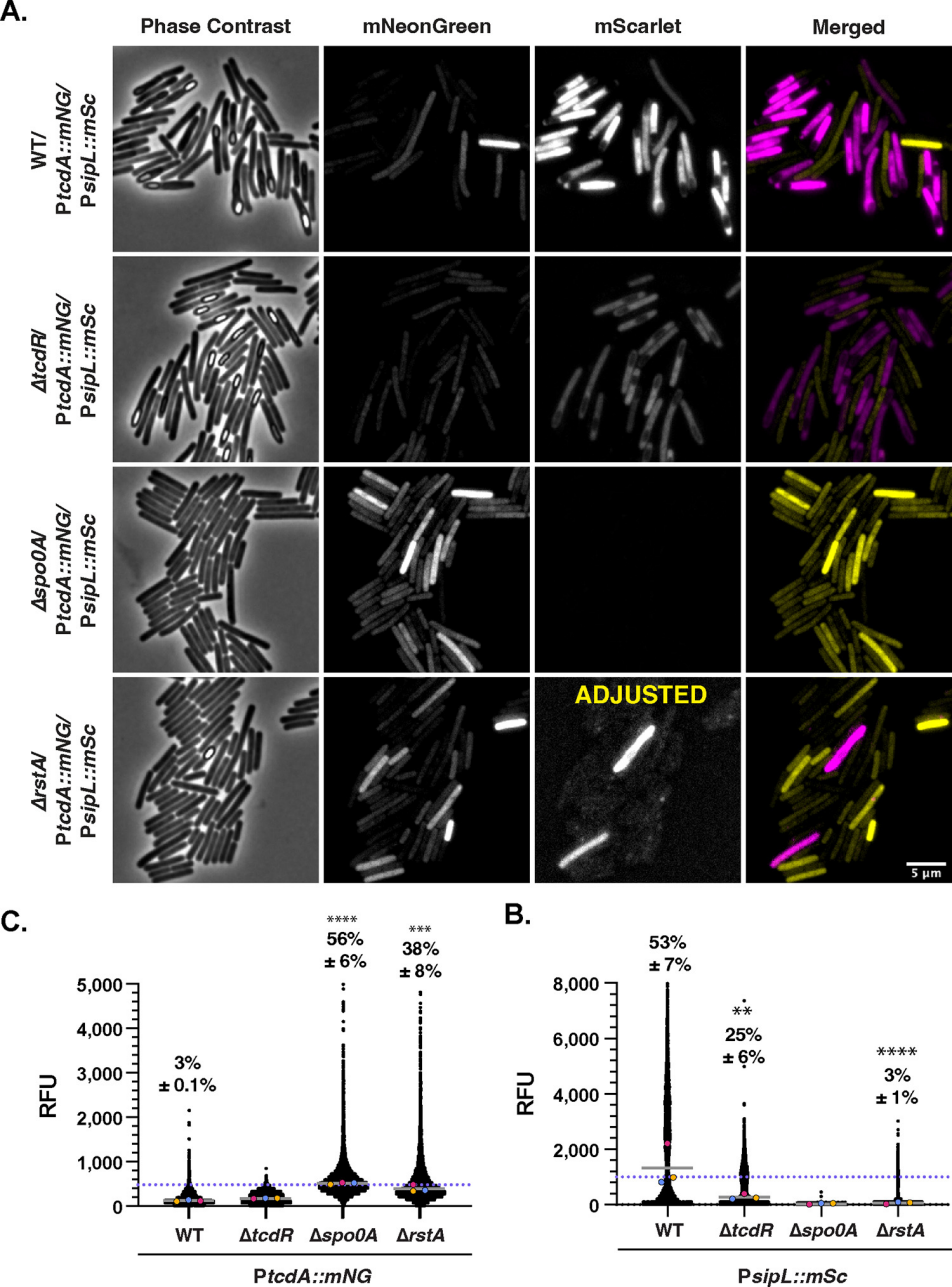

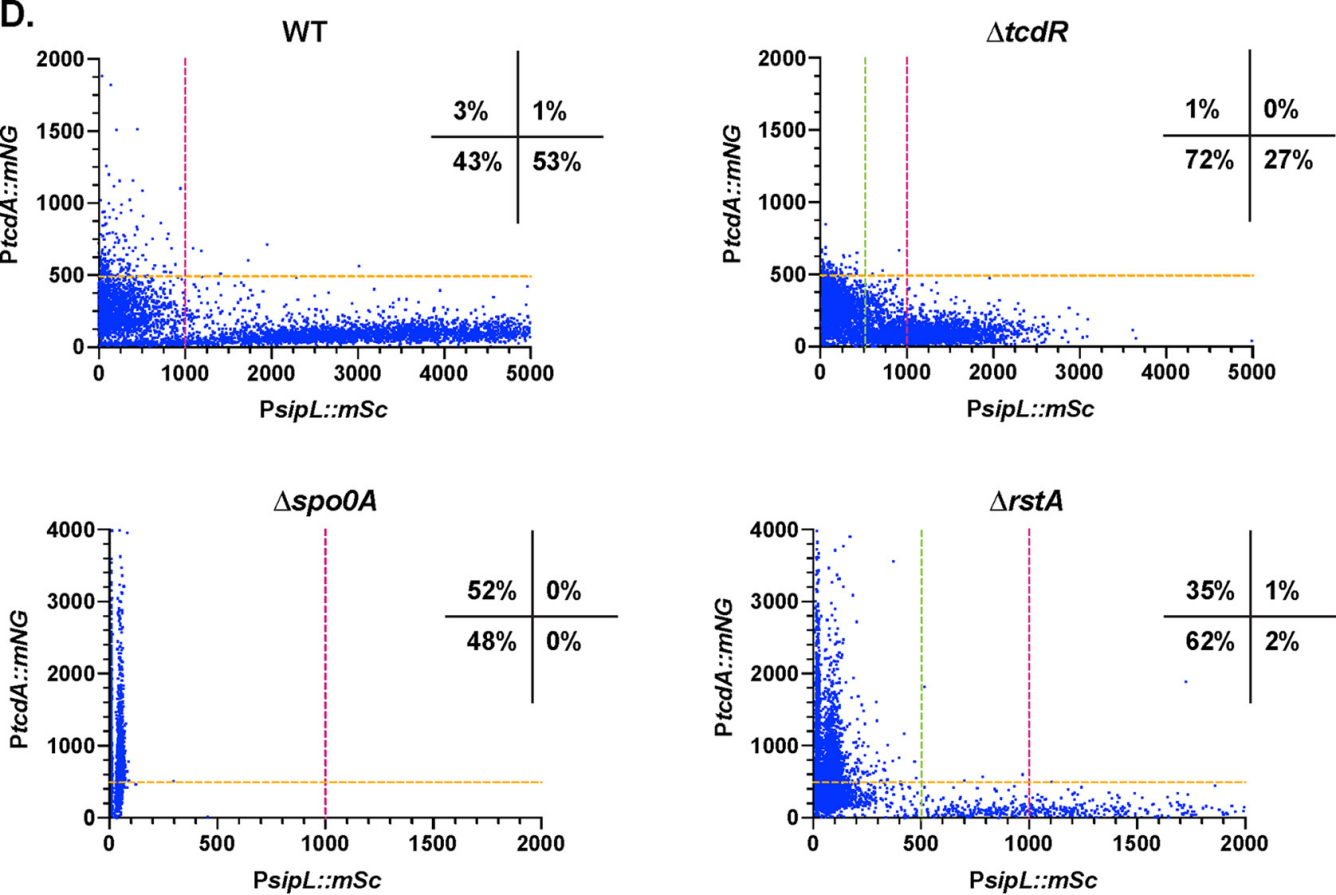
Minimal overlap between toxin and sporulation gene expression is observed during growth on SMC plates. (A) Fluorescence microscopy analyses of fixed bacterial cells after growth on SMC plates. Dual-reporter strains contain P*sipL*::*mScarlet* and P*tcdA*::*mNeonGreen.* Dual-reporter analyses were visualized in WT, Δ*tcdR*, Δ*spo0A*, and Δ*rstA* strain backgrounds. Phase-contrast microscopy was used to visualize all bacterial cells. The merge of mNeonGreen (yellow) with mScarlet (magenta) signals is shown. (B and C) SuperSegger-based quantification of P*tcdA*::*mNG* and P*sipL*::*mSc* reporters shows the mean fluorescence intensity of each cell as a black dot on the scatterplot for the indicated reporters. Individual cell intensities were quantified from three biological replicates, with at least two fields of view per strain per replicate. The magenta, yellow, and blue dots represent the median intensities for the first, second, and third biological replicates, respectively. The gray line represents the mean value of each replicate’s median value. The percent Toxin-ON is displayed, with the standard deviation. Toxin-ON cells were calculated using the 99th percentile of the Δ*tcdR* signal as a cutoff (value displayed as a pink dotted line). The percent Sporulation-ON was calculated using the 1,000-RFU signal as a cutoff (value displayed as a blue dotted line). This cutoff was determined using the histogram analyses in Fig. 5B. A minority of points (<1%) are outside the limits of the scatterplot; axes were adjusted to provide an optimal view of the scatterplot trends. Statistical significance was determined relative to wild-type values using a one-way ANOVA and Tukey’s test. ***, *P* < 0.001; ** *P* < 0.01. (D) Scatterplot analyses show single-cell mean fluorescence intensities with P*sipL*::*mSc* on the *x* axis and *PtcdA*::*mNG* on the *y* axis. Toxin-ON cutoff is represented by the yellow dotted line which indicates 99th percentile of the Δ*tcdR* signal. The Sporulation-ON cutoff is represented by the pink dotted line at 1,000 RFU based on the histogram bimodal distribution analyses; the green line highlights the Sporulation-ON cutoff for Δ*tcdR* and Δ*rstA* based on the histogram analyses.. Percentages indicate Toxin-ON/Sporulation-OFF in the top left quadrant, Toxin-ON/Sporulation-ON in the top right quadrant, Toxin-OFF/Sporulation-OFF in the bottom left quadrant, and Toxin-OFF/Sporulation-ON in the bottom right quadrant. A minority of points (<1%) are outside the limits of the scatterplot; axes were adjusted to provide an optimal view of the scatterplot trends.

While growth on TY agar versus TY broth promoted sporulation by ~3-fold in Δ*tcdR* and Δ*rstA* dual-reporter strains (35% versus 11% for the Δ*tcdR* strain and 6% versus 3% for the Δ*rstA* strain), sporulation in these mutants was still reduced relative to the WT, with Δ*tcdR* and Δ*rstA* strains exhibiting sporulation at ~2- and ~10-fold-lower levels, respectively, than the WT on TY agar. Interestingly, Δ*tcdR* and Δ*rstA* strains also appeared to bifurcate into two distinct subpopulations of toxin and sporulation gene-expressing cells when grown on TY agar versus in TY broth in the scatterplot analyses (Fig. 6 and 7), suggesting that growth on a solid surface may promote a division of labor of toxin versus sporulating gene expression for C. difficile. Notably, growth on TY agar did not change the frequency or magnitude of population-wide Toxin-ON cells detected for Δ*spo0A* and Δ*rstA* cells, with ~80% of Δ*spo0A* and Δ*rstA* cells being designated Toxin-ON when grown on TY agar or plates. Similar trends in terms of the bifurcation in toxin and sporulation gene expression were observed for WT and Δ*tcdR* strains when the dual-reporter strains were grown on SMC sporulation agar (Fig. 8), although this growth condition decreased toxin gene expression frequencies and levels for WT, Δ*spo0A*, and Δ*rstA* strains even further relative to TY agar.

Interestingly, cells that express toxin genes at high levels were less likely to express sporulation genes and vice versa, such that cells that express both toxin and sporulation genes tend to express these genes at low to intermediate levels. This property is most evident in TY broth culture, where wild-type cells were most likely to simultaneously express toxin and sporulation genes. Taken together, our findings indicate that growth on agar plates promotes a division of labor for C. difficile, where specific subsets of cells specialize into toxin gene- versus sporulation gene-expressing cells.

## DISCUSSION

Toxin production and spore formation are critical for C. difficile to cause and transmit disease, respectively, and several regulators that control both these important processes have been identified. While toxin production and spore formation are tightly coordinated in some organisms (10–15), the extent to which C. difficile coordinates these processes remained unclear in the absence of methods for simultaneously visualizing their induction at the single-cell level. In this study, we addressed this question by developing a method for visualizing the expression of two genes of interest using chromosomally encoded mNeonGreen and mScarlet transcriptional reporters in C. difficile. By coupling our dual-reporter system with an automated method for quantifying the expression of these reporters at the single-cell level, we determined that toxin gene expression is generally lower in cells that are sporulating. Mutants that either cannot sporulate (Δ*spo0A*) or sporulate at greatly reduced levels (Δ*rstA*) express toxin genes at higher levels on a per-cell basis and in a greater proportion of cells regardless of the growth conditions tested (Fig. 4 to 8).

However, our data indicate that sporulation and toxin gene expression are not always reciprocally regulated, because a strain that cannot express toxin genes (Δ*tcdR*) expresses sporulation genes at lower levels but with similar frequencies at the single-cell level compared to WT C. difficile (Fig. 4 to 8). Furthermore, growth of C. difficile on agar plates promoted a division of labor between transmission and virulence gene expression even if the medium used for broth culture typically induces toxin gene expression (i.e., TY medium) (Fig. 6 to 8). Interestingly, loss of the RstA regulator in TY broth caused C. difficile to exclusively express toxin genes, whereas 11% of WT cells expressed both toxin and sporulation genes. In contrast, on TY plates, no difference in the frequency between cells expressing both toxin and sporulation genes was observed for WT and Δ*rstA* cells (3 to 4%). How RstA differentially regulates toxin and sporulation gene expression in broth culture versus growth on a surface remains unclear, but in liquid culture, RstA may function to keep a clonal population uncommitted to either sporulation or toxin expression. However, it should be noted that across the multiple growth conditions tested, RstA increased the magnitude of sporulation gene expression and thus skewed the population to induce sporulation. Regardless, our data indicate that C. difficile bifurcates into specialized sporeformers and toxin expressers under certain environmental conditions at least for the 630Δ*erm* strain, although we acknowledge that toxin (18) and sporulation gene expression may be regulated differentially at the single-cell level (36) in other C. difficile strains.

While the physiological importance of this division of labor remains unclear, it can be beneficial for individuals within a bacterial population to specialize and perform specific tasks that benefit the population as a whole (65). This phenomenon has been well documented during biofilm formation (66), where metabolically intensive gene products are produced as “public goods.” For example, B. subtilis biofilms share energy-expensive matrix components with proximal cells (66), and distinct localizations for motile, matrix-producing cells versus sporulating cells are observed (67). Additionally, Pseudomonas aeruginosa was recently shown to use c-di-GMP signaling to divide its “labor” into “biofilm founders,” which produce matrix, and “surface explorers,” which express motility genes (68). Since C. difficile toxin is a metabolically costly secreted product, it may also be utilized as a public good. Indeed, with our toxin gene reporter, we observed that some C. difficile cells express toxin genes at extremely high levels relative to the rest of the population (Fig. 4), and these cells appear less likely to also express the sporulation reporter regardless of growth condition (Fig. 6 and 8). Thus, C. difficile may use a similar strategy to differentiate some cells into high toxin producers so that other cells focus their energy on sporulation or other tasks. Given that our data suggest that C. difficile exhibits a more prominent division of labor when grown on plates than in liquid broth, surface sensing and/or growth in biofilms could play a role in coordinating this specialization. Interestingly, some B. thuringiensis strains subdivide their population into terminally differentiated toxin crystal producers versus spore-formers (16), but the division of labor observed in C. difficile between virulence and transmission gene expression appears to be condition specific. Importantly, a critical question that arises from these analyses in laboratory media is whether C. difficile employs this strategy during infection. During growth in the murine gut, a subpopulation of C. difficile is sporulating, since ~10% of C. difficile cells detected in the gut are present in the spore form (69). However, the proportion of C. difficile cells that are in the process of sporulating during infection is unknown, and current technologies are lacking to sufficiently analyze gene expression at the single-cell level *in vivo*.

Notably, the chromosomal sporulation and toxin gene reporters we have developed could be useful for addressing these questions in the future, especially since our single-cell analyses of toxin and sporulation gene expression in different growth conditions highlight the complex intricacies of transmission and virulence gene expression. Additionally, since C. difficile exhibits considerable genetic heterogeneity (70), an important next step would be to analyze the behavior of these reporters in a variety of C. difficile isolates. For example, toxin gene expression is bimodal in several C. difficile clinical isolates but is less prominent in the 630 strain background (18) used in this study. This is in part because 630 does not phase-vary its flagella because it is locked in the “Flagella-ON” state (71). Since the flagellar switch leads to the production of σ^D^ (22, 40), which upregulates *tcdR* gene expression, it would be useful to analyze our dual sporulation and toxin gene reporter system in a strain like R20291, which undergoes flagellar phase variation (40). These types of analyses may also provide insight into whether the *mNeonGreen* and *mScarlet* reporters are differentially tolerated by diverse C. difficile isolates.

Future studies aimed at investigating how virulence and transmission gene expression are regulated in the host during infection will also be crucial for characterizing C. difficile host-pathogen interactions. Does a division of labor between sporulation and toxin-expression occur in the host? Are toxin-expressing and sporulating subpopulations geographically distinct or stochastically overlapping? Where are transmission and virulence genes expressed, and is there a pattern to their distribution? Utilizing these novel reporters in the context of an infection model could begin to address these important questions and provide mechanistic insight into C. difficile infection.

Finally, the dual-reporter system we have developed opens up numerous possibilities for deciphering the relationship between additional important processes in C. difficile. Recent work has revealed that C. difficile generates phenotypically heterogeneous subpopulations through multiple mechanisms, including bistability in toxin production (18), phase variation in the expression of flagellar genes (40), genes encoding phage receptors (41), and genes for two-component systems (72), and the bimodal expression of sigma factors that respond to stress (42). Having optimized the use of the *mNeonGreen* transcriptional reporter to overcome C. difficile’s natural autofluorescence (provided it is expressed at moderately high levels) and the spectrally compatible *mScarlet* transcriptional reporter, it will now be possible to visualize how C. difficile specializes into different subpopulations and how nutritional inputs or growth conditions affect these different subpopulations. For example, our reporter system could be adapted to visualize how the levels of the important second messenger cyclic di-GMP affect flagellum, toxin, and biofilm production and sporulation (21, 22, 40, 72, 73). Integrating the dual reporters into a single construct would facilitate addressing these questions in a broader range of clinical isolates, since many isolates are challenging to manipulate genetically. These types of dual-reporter analyses will undoubtedly provide important insight into the factors and mechanisms that control phenotypic heterogeneity in C. difficile.

## MATERIALS AND METHODS

### Bacterial strains and growth conditions.

All C. difficile strains used for this study are listed in in Table S1 in the supplemental material. All constructed strains derive from the sequenced clinical isolate 630, but the erythromycin-sensitive 630Δ*ermΔpyrE* is the parental strain used for all strain construction using *pyrE*-based allele-coupled exchange (ACE) (54). Strains were grown on brain heart infusion supplemented (BHIS) with yeast extract and cysteine (74), taurocholate (TA; 0.1% [wt/vol]; 1.9 mM), cefoxitin (8 μg/mL), thiamphenicol (10 to 15 μg/mL), and kanamycin (50 μg/mL) as needed. For ACE, the C. difficile defined medium (CDDM) (75) was supplemented with 5-fluoroorotic acid (5-FOA; 2 mg/mL) and uracil (5 μg/mL).

10.1128/msphere.00132-22.9 TABLE S1 Strains used in this study. Download Table S1, PDF file, 0.2 MB. Copyright © 2022 Donnelly et al. 2022 Donnelly et al. https://creativecommons.org/licenses/by/4.0/ This content is distributed under the terms of the Creative Commons Attribution 4.0 International license.

All C. difficile strains used for microscopy assays were first grown overnight from glycerol stocks on BHIS plates supplemented with TA (0.1% [wt/vol]). For broth culture microscopy assays, overnight cultures were inoculated from the BHIS-TA plates and grown in either BHIS or tryptone yeast (TY) broth (76). For plate-based microscopy assays, C. difficile strains were inoculated from BHIS-TA plates into BHIS liquid medium and grown until they were turbid. The cultures were then back-diluted 1:25 until they reached an optical density at 600 nm (OD_600_) between 0.4 and 0.7. One hundred twenty microliters of this mid-log-phase culture was used to inoculate either TY plates or SMC plates (77) for 18 h prior to imaging.

Escherichia coli strains used for HB101/pRK24-based conjugations are listed in Table S1. E. coli strains were grown at 37°C with shaking at 225 rpm in Luria-Bertani (LB) broth. The medium was supplemented with ampicillin (50 μg/mL) and chloramphenicol (20 μg/mL) as needed.

### E. coli strain construction.

All primers used for cloning are listed in Table S2 and plasmid maps for each construct are provided in Table S2. All plasmid constructs were sequence-confirmed using Sanger sequencing through Genewiz. Plasmids were transformed into HB101/pRK24 E. coli and subsequently used to conjugate sequence-confirmed plasmids into C. difficile.

10.1128/msphere.00132-22.10 TABLE S2 Primers used in this study. Download Table S2, PDF file, 0.1 MB. Copyright © 2022 Donnelly et al. 2022 Donnelly et al. https://creativecommons.org/licenses/by/4.0/ This content is distributed under the terms of the Creative Commons Attribution 4.0 International license.

### C. difficile strain construction.

Δ*tcdR* and Δ*rstA* deletion strains were generated using ACE (54) with pMTL-YN3-Δ*tcdR* and pMTL-YN3-Δ*rstA*, respectively, and the parental 630Δ*erm*Δ*pyrE* strain. Single-reporter strains and complementation strains were generated as previously described by conjugating HB101/pRK24-carrying pMTL-YN1C plasmids into Δ*pyrE*-based strains (78) using ACE. To generate dual-reporter strains, P*sipL*::*mScarlet* was introduced downstream of the *sipL* locus of 630Δ*erm*Δ*pyrE*, 630Δ*ermΔtcdR*Δ*pyrE*, 630Δ*ermΔspo0A*Δ*pyrE*, and 630Δ*ermΔrstA*Δ*pyrE* using ACE and pMTL-YN3-P*sipL*::*mScarlet*. The second reporter, P*tcdA*::*mNeonGreen*, was then introduced into the *pyrE* locus of the resulting strains using pMTL-YN1C-P*tcdA*::*mNeonGreen*. At least two clones of each strain generated by allelic exchange were phenotypically characterized prior to restoration of the *pyrE* locus using pMTL-YN1C. At least two clones of every complementation strain were integrated into the *pyrE* locus and phenotypically characterized.

### Growth curves.

C. difficile cultures were grown in 2 mL BHIS medium to stationary phase (4 to 5 h) and then were back-diluted 1:25 in 2.5 mL BHIS until an OD_600_ of 0.5 was obtained. All strains were normalized to an OD_600_ of 0.5 if growth rates varied. Approximately 3.5 × 10^5^ CFU were inoculated into 150 μL of the indicated media (~50 μL of the OD_600_ 0.5 culture into 150 μL). One hundred fifty microliters of each strain was added to a 96-well plate (in technical triplicate) alongside appropriate blanks, and the plates were sealed with clear, gas-permeable enzyme-linked immunosorbent assay (ELISA) plate sealers (R&D Systems). Plates were read in an Epoch plate reader (BioTek) in the anaerobic chamber with OD_600_ readings performed every 15 min after a 2-min linear shake.

### mScarlet maturation assay.

For bulk fluorophore maturation quantification, replicate 96-well plates were set up as described above for the growth curves. Sealed plates were incubated overnight with gentle shaking in the anaerobic chamber. After overnight growth, plates were removed from the chamber, the seal was removed to expose cells to oxygen, and then the plates were read in a Synergy H1 (BioTek) plate reader under ambient conditions at 37°C, with readings performed every 4 min for 24 h, with orbital shaking every 5 s. The mScarlet fluorophore was excited at 560 nm, and its emission was detected at 600 nm. This assay was performed in three biological replicates, with two technical replicates randomly selected to be included in Fig. 2.

### Fixation protocol.

Cells were fixed as previously described (43, 79). In brief, a 500-μL aliquot of cells grown in TY broth (or ~1/2 loop of cells grown on plates resuspended in 500 μL TY broth) was added directly to a tube containing 120 μL of a 5× fixation cocktail (100 μL of 16% [wt/vol] paraformaldehyde aqueous solution [methanol-free] [Alfa Aesar]) and 20 μL of 1 M NaPO_4_ buffer (pH 7.4). The samples were mixed and incubated aerobically for 30 min at room temperature in the dark followed by 30 min on ice in the dark. The fixed cells were washed 3 times in phosphate-buffered saline (PBS) and resuspended in 500 μL to 1 mL of PBS (depending on the density of the culture). Cells were immobilized on agarose pads, and slides were incubated at 37°C for at least 2 h to allow mScarlet fluorophore maturation.

### Fluorescence microscopy.

Bacterial cells were immobilized by spotting 1 μL of bacterial culture onto a 1.5% agar pad. Slides were incubated at 37°C for adequate maturation time (2 h for mScarlet for live cell imaging) (Fig. 2). Nucleoid was stained using Hoechst 33342 (15 μg/mL; Molecular Probes). All images were acquired using a Leica DMi8 Thunder imager equipped with an HC PL APO 63×/1.4 numerical aperture (NA) phase-contrast oil immersion objective. Excitation light was generated by a Lumencor Spectra-X multi-LED light source with integrated excitation filters. For all fluorescent channels aside from YFP, an XLED-QP quadruple-band dichroic beam-splitter (Leica) was used (transmission: 415, 470, 570, and 660 nm) along with an external filter wheel (Leica). Phase-contrast images were taken with a 50-ms exposure time. Hoechst was excited at 395/40 nm (15% intensity) with a 100-ms exposure, and emitted light was filtered using a 440/40-nm emission filter (Leica). mScarlet was excited at 555/38 nm (20% intensity) with 150 ms exposure time, and emitted light was filtered using a 590/50-nm emission filter (Leica). mNeonGreen images were captured with a YFP filter set (Chroma) equipped with a 500/20-nm excitation filter, a 515-nm dichroic filter, and a 535/30-nm emission filter; excitation light was generated using the 510-nm LED line (100% intensity) and 387 ms exposure. Emitted and transmitted light was detected using a Leica DFC 9000 GTC sCMOS camera. Three- to 4-μm Z-stacks were taken for each strain with 0.213-μm z-slices. All strains for a given experiment (6 to 8 strains) were spotted and captured sequentially on the same agar pad, and Leica Adaptive Focus Control hardware autofocus was used to maintain focus at each position throughout the acquisition.

### Image analysis and quantification.

After image acquisition, images were processed using Leica Instant Computational Clearing (ICC) applied to the fluorescent channels to avoid bleed-through of fluorescent signal into neighboring cells. The adaptive strategy was run with the feature scale set to 2,683 nm and 98% strength. Following ICC, images were exported from the LASX software (Leica) and further processed using FIJI. Following export, images were cropped to remove out-of-focus regions and the best-focused Z-plane was selected for each channel to correct for chromatic aberration using FIJI. Images were quantitatively analyzed using the SuperSegger pipeline (55) in MATLAB with the supplied 60× analysis settings. The output clist matrices containing all single-cell data were exported from MATLAB, and the mean intensity was plotted as a scatterplot and/or histogram using GraphPad Prism (version 9.0.2). Images were also analyzed manually in FIJI by examining the fluorescence intensities of visibly sporulating cells to confirm that the cutoffs identified based on histogram analyses for Sporulation-ON were consistent with this visual inspection. Image scaling was adjusted to improve brightness and contrast for display and was applied equally to all strains in each experiment unless otherwise indicated by “ADJUSTED” on the image. While some images are displayed with some saturation in the signal (clipping), no clipping occurred during image acquisition or quantification. At least three images per strain were captured in each replicate and every strain was analyzed with three biological replicates. Image analysis was performed on at least two positions per replicate. Statistical significance was determined using one-way analysis of variance (ANOVA) and Tukey’s test comparing the mean value of each replicate’s median value of three biological replicates (80).

### Ethanol resistance sporulation assay.

C. difficile cultures were grown in BHIS for 3 to 4 h and back-diluted 1:25, and after an OD_600_ of 0.35 to 0.7 was obtained, 120 μL of the log-phase culture was plated on 70:30 sporulation agar to form a lawn (77). Ethanol resistance was used to determine the sporulation efficiency of a given strain based on previously described procedures (81, 82). Specifically, after 24 h on 70:30 medium, cells were resuspended in 70:30 broth to an OD_600_ of 1.0. Cells were immediately serially diluted in 70:30 broth and plated on BHIS medium with 0.1% taurocholate to enumerate all viable vegetative cells and spores. A 0.5-mL aliquot of culture was removed from the chamber, mixed with 0.5 mL 95% ethanol, subjected to vortex mixing, incubated at room temperature for 15 min, serially diluted in 1× PBS, and plated on BHIS with 0.1% taurocholate to enumerate spores. After 24 h growth, CFU were enumerated, and the sporulation frequency was calculated as the number of ethanol-resistant spores divided by the total number of viable cells. The average ratio of ethanol-resistant CFU obtained from functional spores for a given strain relative to the average ratio determined for the wild type was determined from a minimum of three biological replicates. A *spo0A* mutant was used as a negative control. Statistical significance was determined using one-way ANOVA and Tukey’s test.

### Western blotting.

Samples for Western blot analyses were prepared as previously described (77), with minor adjustments. C. difficile was grown overnight for 18 h in TY broth to induce toxin expression. For each strain, 1 mL of culture was pelleted and resuspended in 50 μL of PBS, and samples were freeze-thawed for three cycles prior to resuspension in 100 μL of EBB buffer (8 M urea, 2 M thiourea, 4% [wt/vol] SDS, 2% [vol/vol] β-mercaptoethanol). The samples were boiled for 20 min, subjected to vortex mixing, pelleted at high speed, and resuspended in the existing buffer to maximize protein solubilization. Finally, the samples were boiled again for 5 min and pelleted at high speed, and FSB (final sample buffer) was added. Samples were resolved on 7.5% SDS-PAGE gels and then transferred to an Immobilon-FL polyvinylidene difluoride (PVDF) membrane. The membranes were blocked in Odyssey blocking buffer with 0.1% (vol/vol) Tween20. Mouse anti-*tcdA* antibody was used at a 1:1,000 dilution (a kind gift from D. Borden Lacy), and chicken anti-glutamate dehydrogenase (GDH) antibody was used at a 1:5,000 dilution as a loading control. IRDye 680CW and 800CW infrared dye-conjugated secondary antibodies were used at a 1:20,000 dilution, and blots were imaged on an Odyssey LiCor CLx imaging system.

### Quantification of Western blotting.

TcdA levels from Western blot analyses of three biological replicates were quantified using LiCor ImageStudio software and normalized to the WT value using the sum-of-all-data-points method (83). Statistical significance was determined using one-way ANOVA and Tukey’s test.
