# Supplementary figures and images for: Development of a Dual-Fluorescent-Reporter System in Clostridioides difficile Reveals a Division of Labor between Virulence and Transmission Gene Expression
Source: mSphere. 2022 May 31;7(3):e00132-22. doi: 10.1128/msphere.00132-22 (PMC9241537; doi:10.1128/msphere.00132-22)

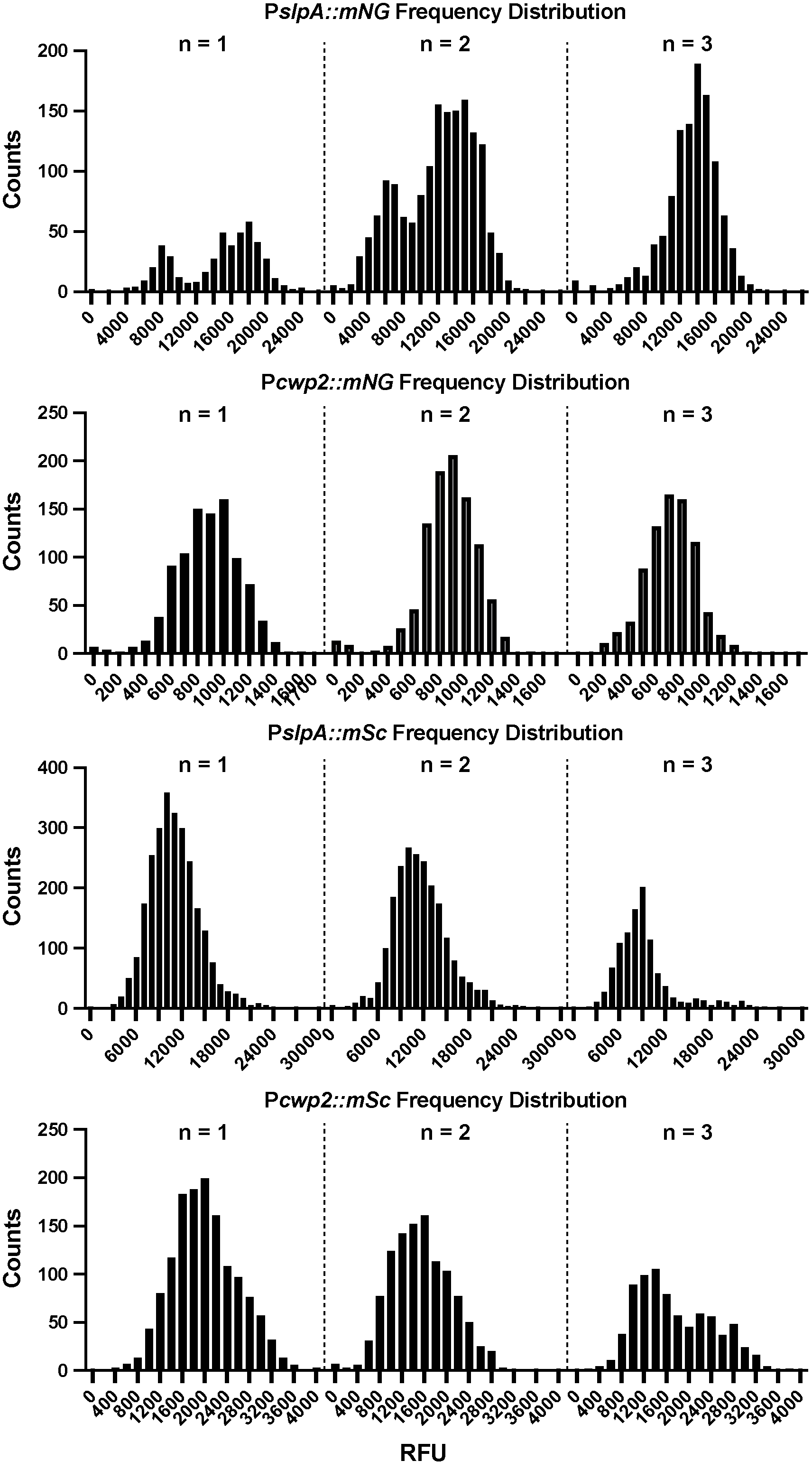

Supplement: FIG S1 [file msphere.00132-22-s0001.tif]

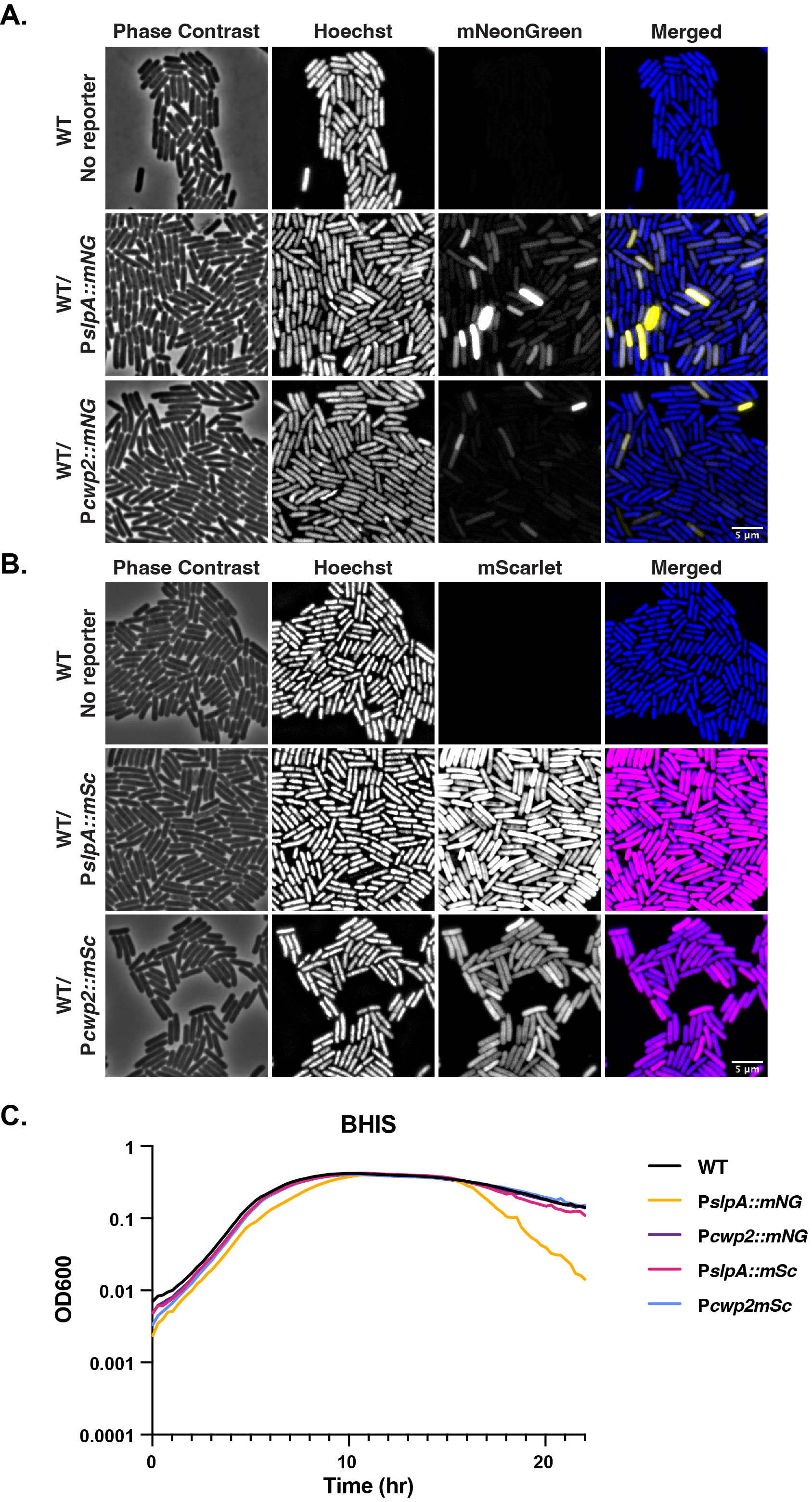

Supplement: FIG S2 [file msphere.00132-22-s0002.jpg]

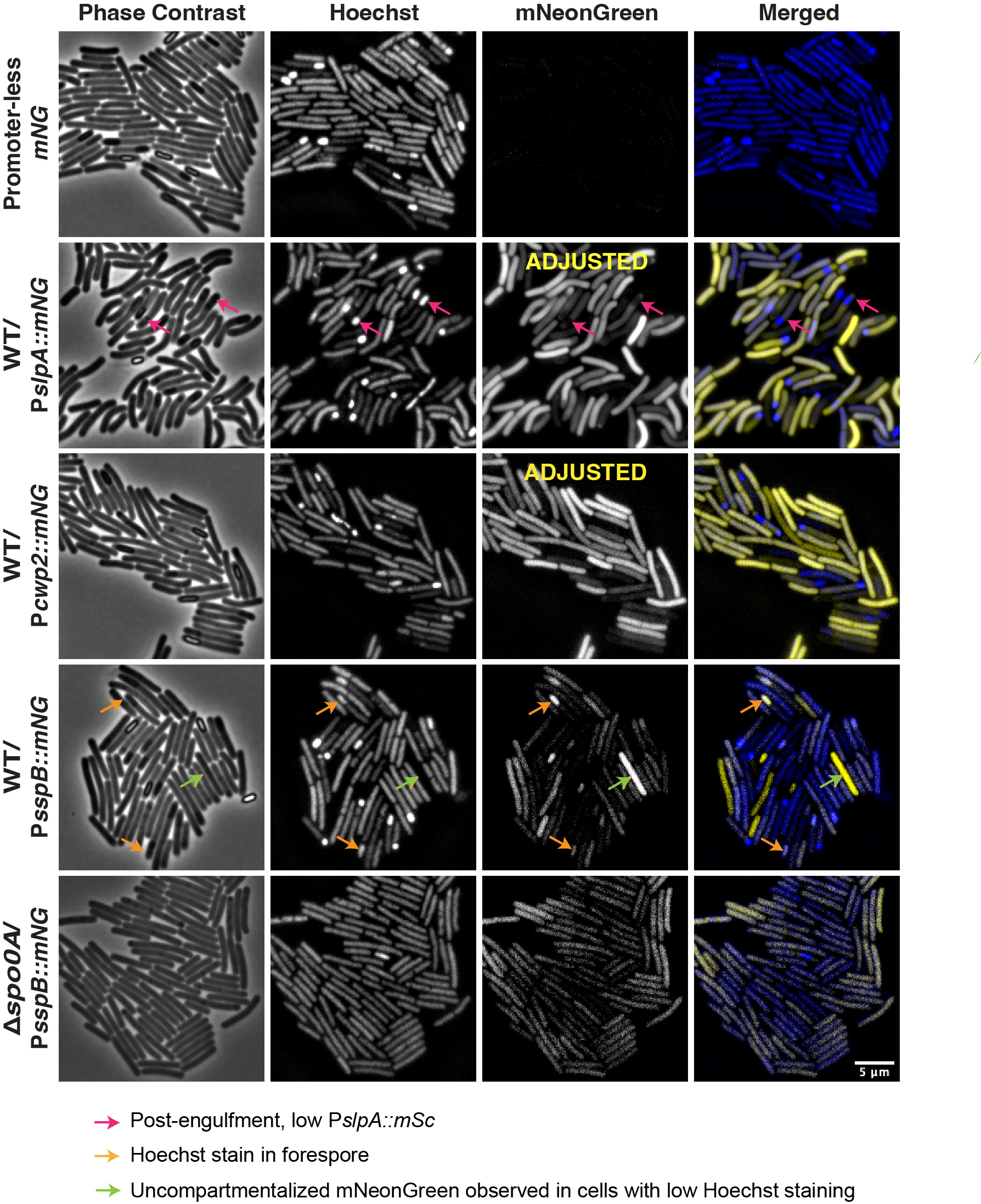

Supplement: FIG S3 [file msphere.00132-22-s0003.jpg]

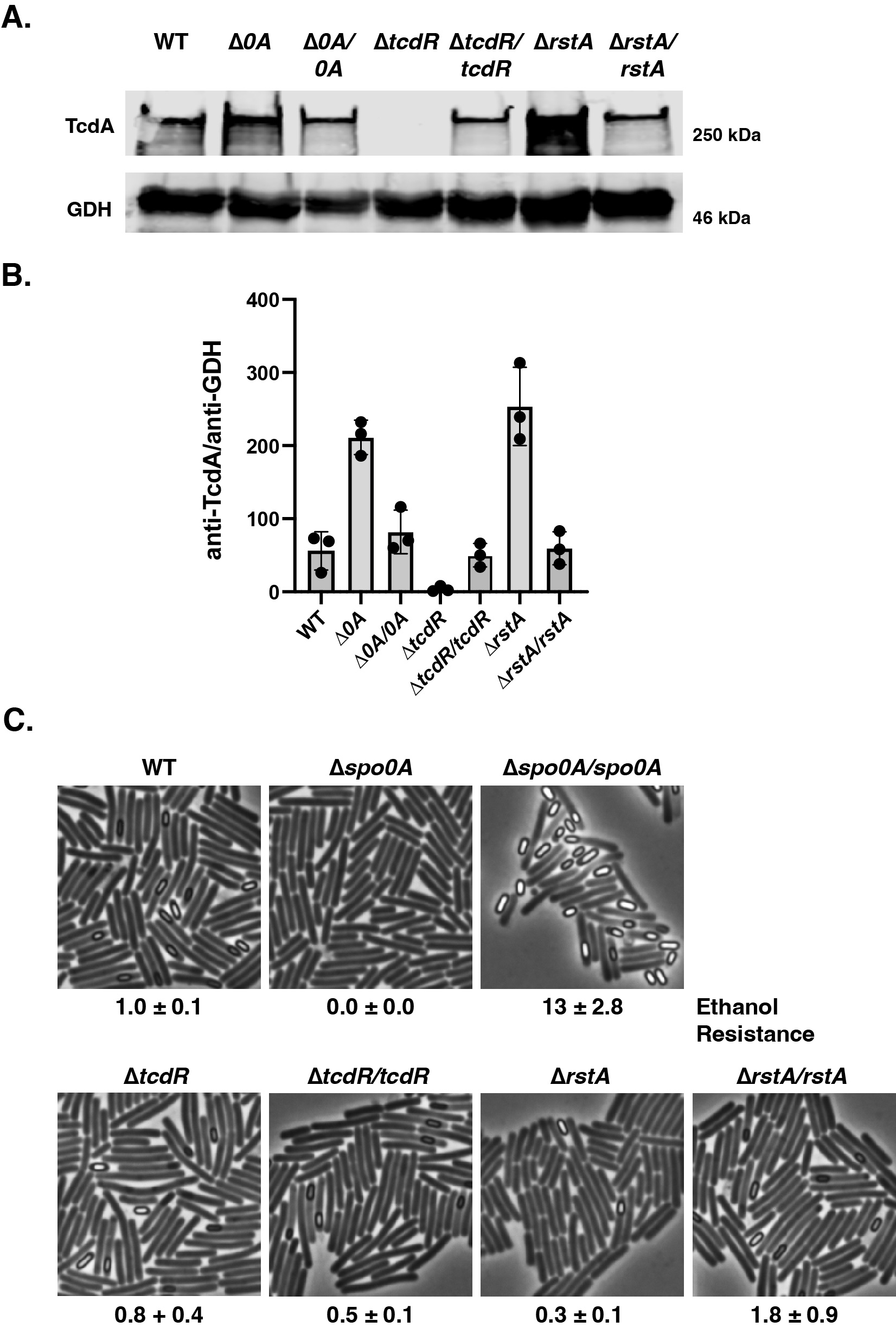

Supplement: FIG S4 [file msphere.00132-22-s0004.jpg]

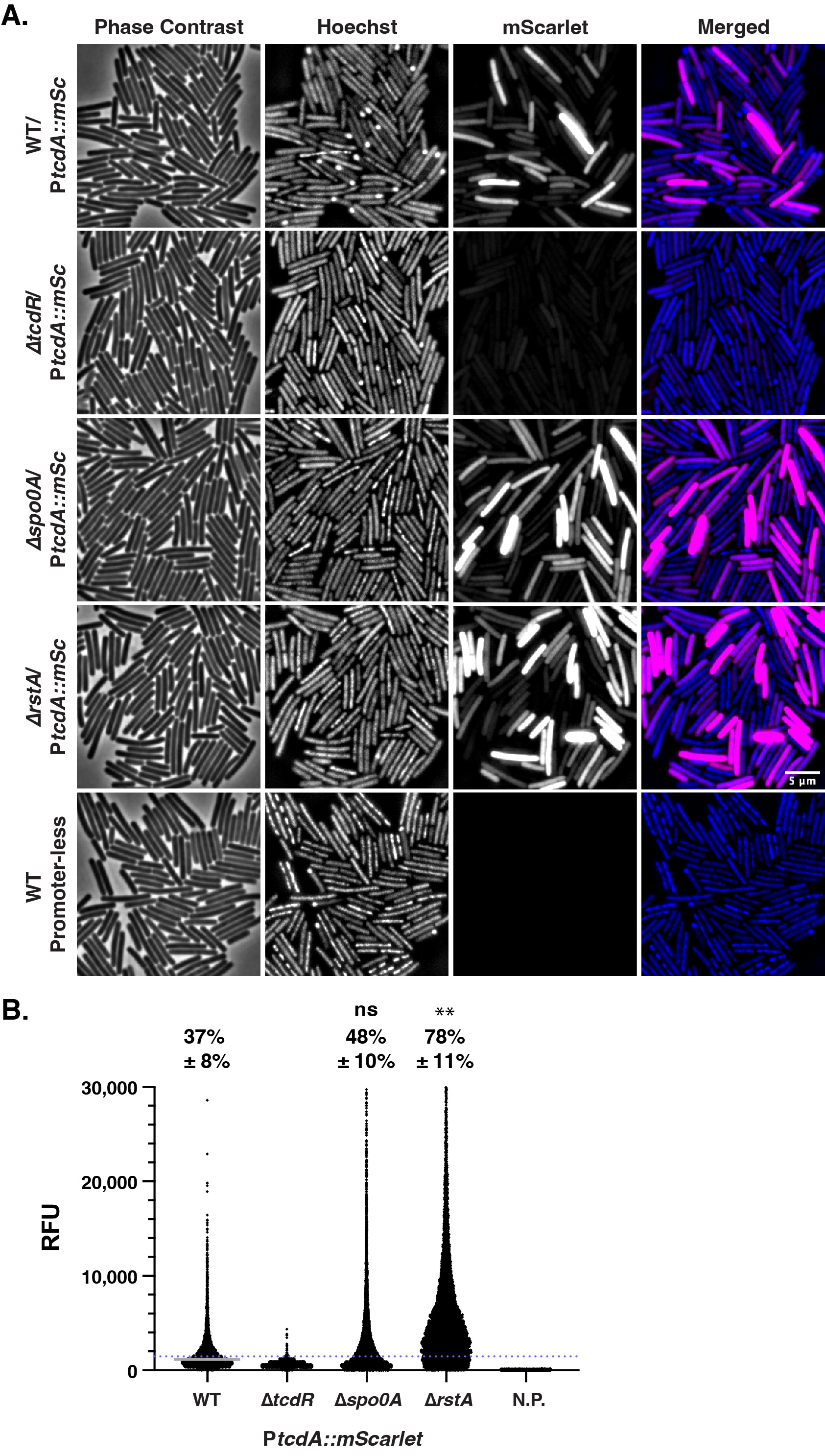

Supplement: FIG S5 [file msphere.00132-22-s0005.jpg]

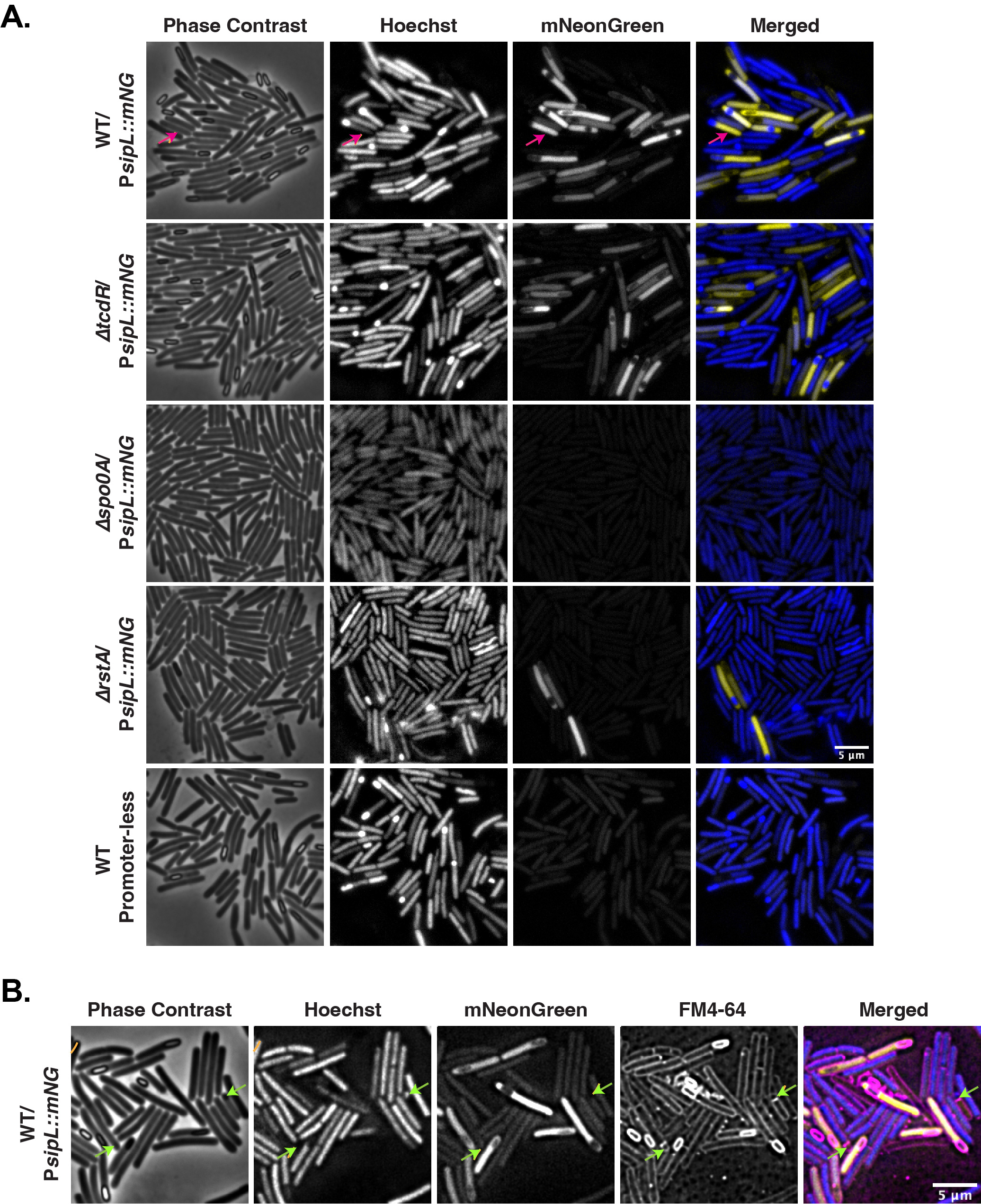

Supplement: FIG S7 [file msphere.00132-22-s0007.jpg]

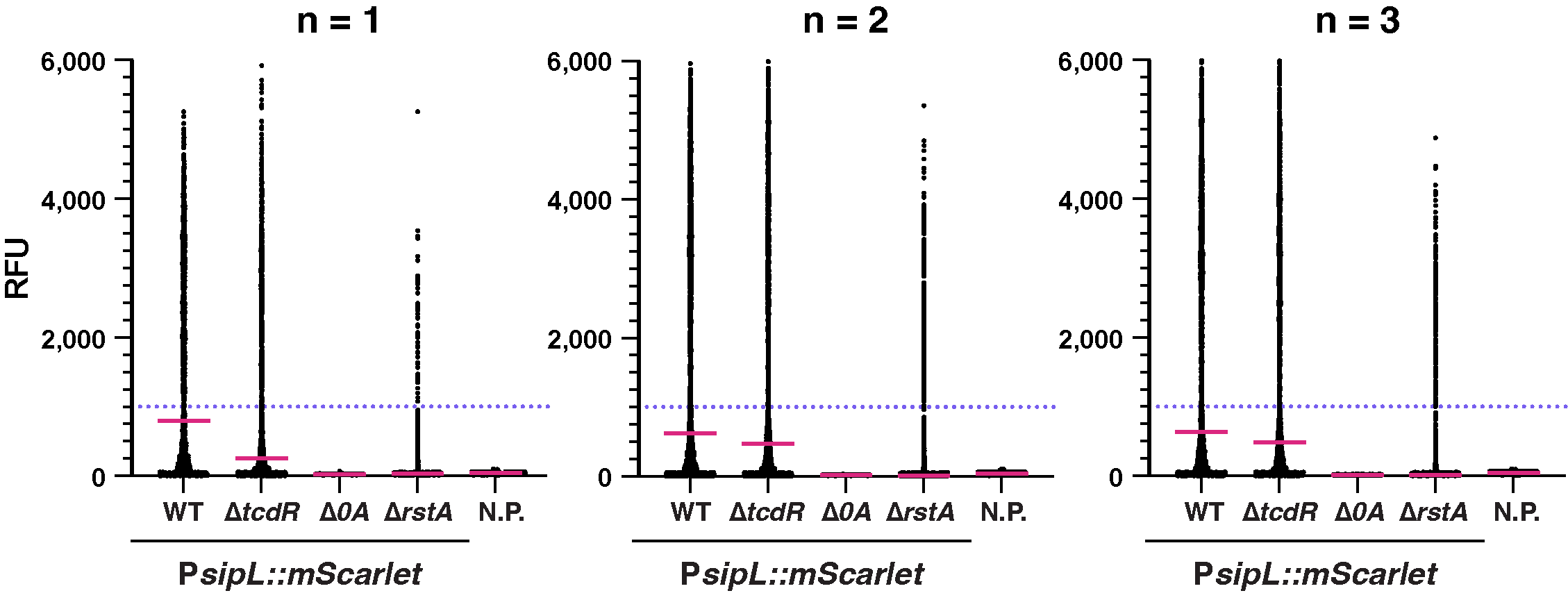

Supplement: FIG S8 [file msphere.00132-22-s0008.tif]
