# Supplementary material for: Development of a Dual-Fluorescent-Reporter System in Clostridioides difficile Reveals a Division of Labor between Virulence and Transmission Gene Expression
Source: mSphere. 2022 May 31;7(3):e00132-22. doi: 10.1128/msphere.00132-22 (PMC9241537; doi:10.1128/msphere.00132-22)

C.

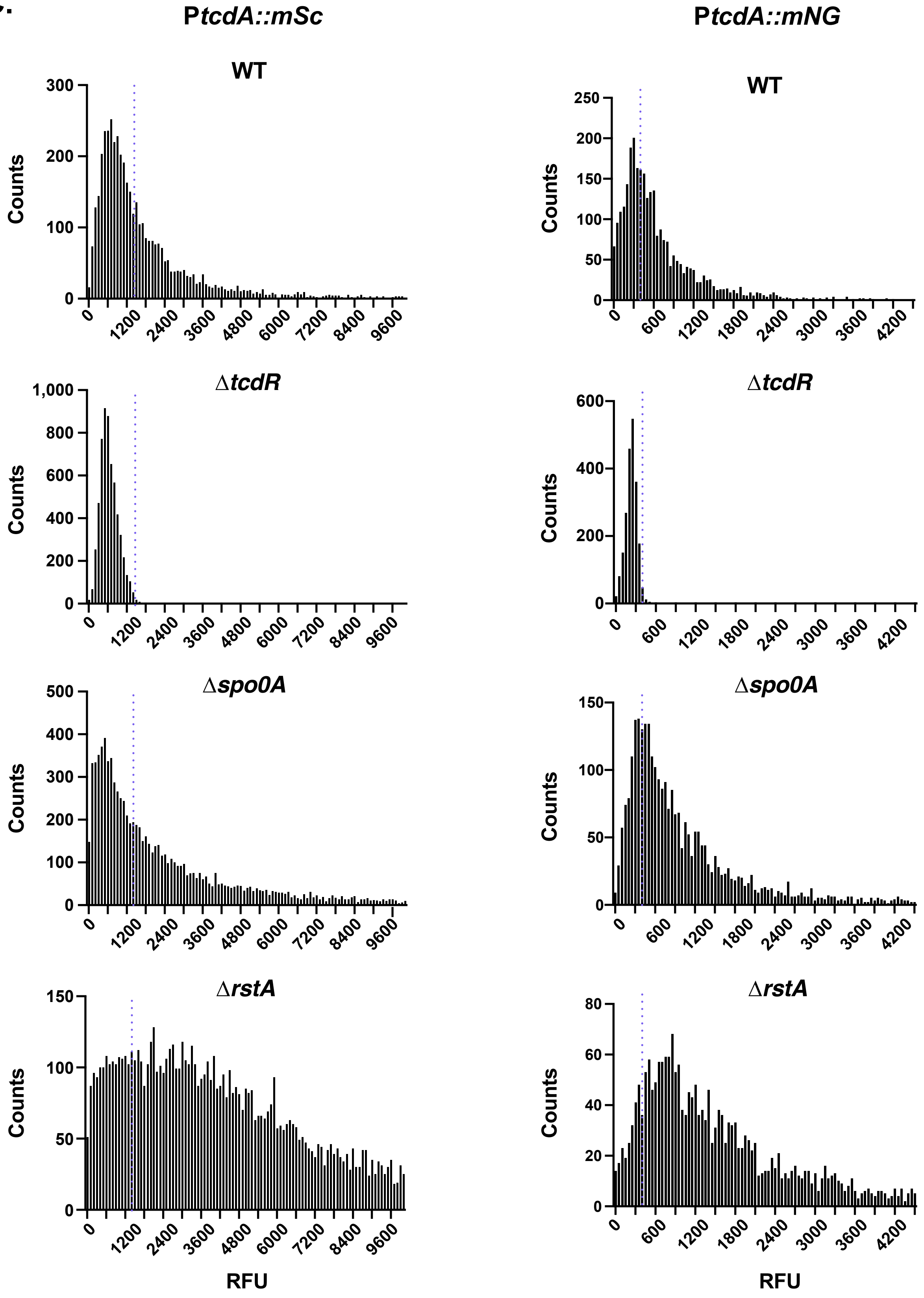
PtcdA::mSc
PtcdA::mNG
WT
ΔtcdR
Δspo0A
ΔrstA
Counts
RFU
0
1200
2400
3600
4800
6000
7200
8400
9600
600
1800
3000
4200
100
200
300
1,000
400
500
800
50
150
250
20
40
60
80

Supplemental Figure 6. Triplicate analyses of toxin gene reporters in TY broth.

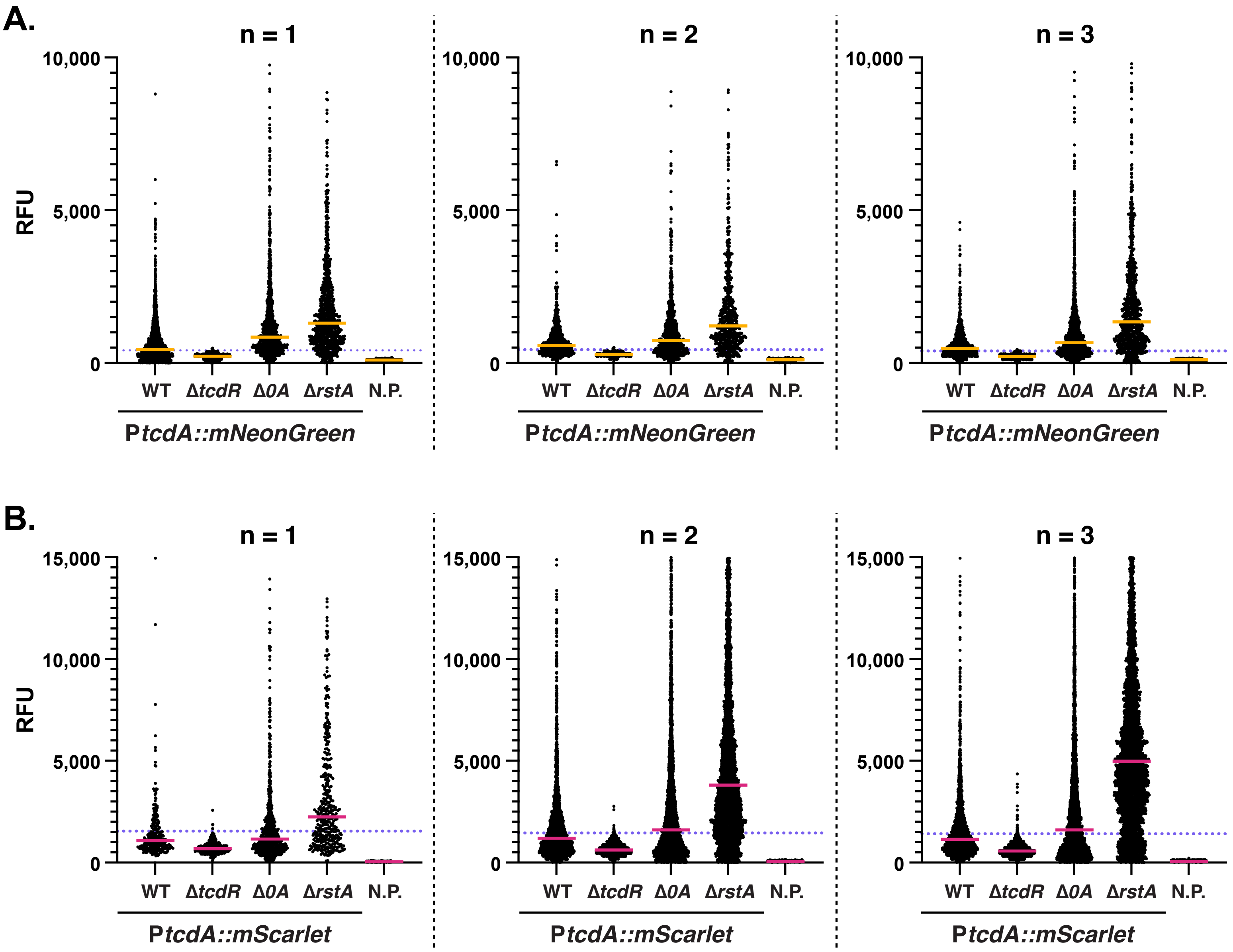

Supplement: FIG S6 [file msphere.00132-22-s0006.pdf]
