# Supplementary material for: Development of a Dual-Fluorescent-Reporter System in Clostridioides difficile Reveals a Division of Labor between Virulence and Transmission Gene Expression
Source: mSphere. 2022 May 31;7(3):e00132-22. doi: 10.1128/msphere.00132-22 (PMC9241537; doi:10.1128/msphere.00132-22)
Supplement: TABLE S1 [file msphere.00132-22-s0009.pdf]

Table S1. Strains used in this study

C. difficile strains – 630Δ*erm*

Strain #	Strain name	Relevant genotype or features	Source/reference
846	630Δ*erm*-p	*erm*-sensitive derivate of 630 with *pyrE* restored	(12)
849	630Δ*erm* Δ*spo0A*-p	630Δ*erm* Δ*spo0A* with *pyrE* restored	(12)
787	630Δ*erm* Δ*pyrE* Δ*spo0A*	630Δ*erm* Δ*pyrE* with *spo0A* deleted	(2)
846	630Δ*erm*-p	*erm*-sensitive derivate of 630 with *pyrE* restored	(2)
849	630Δ*erm* Δ*spo0A*-p	630Δ*erm* Δ*spo0A* with *pyrE* restored	(2)
852	630Δ*erm* Δ*spo0A*/*spo0A*	630Δ*erm* Δ*spo0A* with *spo0A* in the *pyrE* locus	This study
2413	630Δ*erm*/ P*slpA*::*mNeonGreen*	630Δ*erm* with P*slpA::mNeonGreen* in the *pyrE* locus	This study
2417	630Δ*erm*/ P*slpA*::*mScarlet*	630Δ*erm* with P*slpA::mScarlet* in the *pyrE* locus	This study
2627	630Δ*erm*/ P*sspB*::*mScarlet*	630Δ*erm* with P*sspB::mScarlet* in the *pyrE* locus	This study
2630	630Δ*erm*/ P*sspB*::*mNeonGreen*	630Δ*erm* with P*sspB::mNeonGreen* in the *pyrE* locus	This study
2647	630Δ*erm*/ P*tcdA*::*mNeonGreen*	630Δ*erm* with P*tcdA::mNeonGreen* in the *pyrE* locus	This study
2650	630Δ*erm*/ P*tcdA*::*mScarlet*	630Δ*erm* with P*tcdA::mScarlet* in the *pyrE* locus	This study
2683	*630Δerm* Δ*pyrE* Δ*tcdR*	630Δ*erm* Δ*pyrE* with *tcdR* deleted	This study
2686	630Δ*erm* Δ*spo0A*/ P*sspB::mScarlet*	630Δ*erm* Δ*spo0A* with P*sspB::mScarlet* in the *pyrE* locus	This study
2689	630Δ*erm* Δ*spo0A*/ P*sspB::mNeonGreen*	630Δ*erm* Δ*spo0A* with P*sspB::mNeonGreen* in the *pyrE* locus	This study
2752	630Δ*erm*/ P*sipL*::*mScarlet*	630Δ*erm* with P*sipL::mScarlet* in the *pyrE* locus (with the native *sipL* ribosome binding site)	This study
2809	630Δ*erm* Δ*tcdR*/ P*tcdA*::*mNeonGreen*	630Δ*erm* Δ*tcdR* with P*tcdA::mNeonGreen* in the *pyrE* locus	This study
2812	630Δ*erm* Δ*tcdR*/ P*tcdA*::*mScarlet*	630Δ*erm* Δ*tcdR* with P*tcdA::mScarlet* in the *pyrE* locus	This study
2821	630Δ*erm*/ *mScarlet*	630Δ*erm* with *mScarlet* without a promoter (but with the *slpA* RBS) in the *pyrE* locus	This study
2824	630Δ*erm*/ *mNeonGreen*	630Δ*erm* with *mNeonGreen* without a promoter (but with the *slpA* RBS) in the *pyrE* locus	This study
3471	630Δ*erm* Δ*tcdR*/ *tcdR*	630Δ*erm* Δ*tcdR* with *tcdR* in the *pyrE* locus	This study
3491	630Δ*erm* Δ*pyrE* Δ*rstA*	630Δ*erm* Δ*pyrE* with *rstA* deleted	This study
3537	630Δ*erm* Δ*rstA*/ P*tcdA*::*mScarlet*	630Δ*erm* Δ*rstA* with P*tcdA::mScarlet* in the *pyrE* locus	This study
3543	630Δ*erm* Δ*rstA*/ P*sipL*::*mScarlet*	630Δ*erm* Δ*rstA* with P*sipL::mScarlet* in the *pyrE* locus	ΔrstA
3587	630Δ*erm* Δ*spo0A*/ P*sipL*::*mScarlet*	630Δ*erm* Δ*spo0A* with P*sipL::mScarlet* in the *pyrE* locus	This study
3807	630Δ*erm* Δ*rstA*/ *rstA*	630Δ*erm* Δ*rstA* with *rstA* in the *pyrE* locus	This study
3823	630Δ*erm*/ P*cwp2*::*mScarlet*	630Δ*erm* with P*cwp2::mScarlet* in the *pyrE* locus	This study
3826	630Δ*erm*/ P*cwp2*::*mNeonGreen*	630Δ*erm* with P*cwp2::mNeonGreen* in the *pyrE* locus	This study
3840	630Δ*erm* Δ*tcdR*-p	630Δ*erm* Δ*tcdR* with *pyrE* restored	This study
3843	630Δ*erm* Δ*rstA*-p	630Δ*erm* Δ*rstA* with *pyrE* restored	This study
3846	630Δ*erm* Δ*tcdR*/ P*sipL*::*mScarlet*	630Δ*erm* Δ*tcdR* with P*sipL::mScarlet* in the *pyrE* locus	This study
3862	630Δ*erm* Δ*spo0A*/ P*tcdA*::*mScarlet*	630Δ*erm* Δ*spo0A* with P*tcdA::mScarlet* in the *pyrE* locus	This study
3865	630Δ*erm* Δ*spo0A*/ P*tcdA*::*mNeonGreen*	630Δ*erm* Δ*spo0A* with P*tcdA::mNeonGreen* in the *pyrE* locus	This study
3868	630Δ*erm* Δ*pyrE* Δ*rstA*/ P*sipL*::*mScarlet*	630Δ*erm* Δ*pyrE* Δ*rstA* with P*sipL::mScarlet* in the *sipL* locus	This study
3870	630Δ*erm* Δ*pyrE*/ P*sipL*::*mScarlet*	630Δ*erm* Δ*pyrE* with P*sipL::mScarlet* in the *sipL* locus	This study
3873	630Δ*erm* Δ*pyrE* Δ*tcdR*/ P*sipL*::*mScarlet*	630Δ*erm* Δ*pyrE* Δ*tcdR* with P*sipL::mScarlet* in the *sipL* locus	This study
3877	630Δ*erm* Δ*tcdR*/ P*sipL*::*mScarlet*/ P*tcdA::mNeonGreen*	630Δ*erm* Δ*tcdR* with P*sipL::mScarlet* in the *sipL* locus and P*tcdA::mNeonGreen* in the *pyrE* locus	This study
3880	630Δ*erm* Δ*rstA*/ P*sipL*::*mScarlet*/ P*tcdA::mNeonGreen*	630Δ*erm* Δ*rstA* with P*sipL::mScarlet* in the *sipL* locus and P*tcdA::mNeonGreen* in the *pyrE* locus	This study

3883	630Δ*erm*/ P*sipL*::*mNeonGreen*	630Δ*erm* with P*sipL::mNeonGreen* in the *pyrE* locus	This study
3889	630Δ*erm* Δ*rstA*/ P*tcdA*::*mNeonGreen*	630Δ*erm* Δ*rstA* with P*tcdA::mNeonGreen* in the *pyrE* locus	This study
3892	630Δ*erm* P*sipL*::*mScarlet*/ P*tcdA*::*mNeonGreen*	630Δ*erm* with P*sipL::mScarlet* in the *sipL* locus and P*tcdA::mNeonGreen* in the *pyrE* locus	This study
3895	630Δ*erm* Δ*tcdR*/ P*sipL*::*mNeonGreen*	630Δ*erm* Δ*tcdR* with P*sipL::mNeonGreen* in the *pyrE* locus	This study
3898	630Δ*erm* Δ*rstA*/ P*sipL*::*mNeonGreen*	630Δ*erm* Δ*rstA* with P*sipL::mNeonGreen* in the *pyrE* locus	This study
3901	630Δ*erm* Δ*spo0A*/ P*sipL*::*mNeonGreen*	630Δ*erm* Δ*spo0A* with P*sipL::mNeonGreen* in the *pyrE* locus	This study
3904	630Δ*erm* Δ*spo0A*/ P*sipL*::*mScarlet*	630Δ*erm* Δ*pyrE* Δ*spo0A* with P*sipL::mScarlet* in the *sipL* locus	This study
3907	630Δ*erm* Δ*spo0A*/ P*sipL*::*mScarlet*/ P*tcdA*::*mNeonGreen*	630Δ*erm* Δ*spo0A* with P*sipL::mScarlet* in the *sipL* locus and P*tcdA::mNeonGreen* in the *pyrE* locus	This study

HB101 *E. coli* strains

***E. coli* strains**			
**Strain #**	**Strain name**	**Benchling plasmid map with primers**	**Source**
41	DH5α	F– Φ80*lacZ*ΔM15 Δ(*lacZYA-argF*) U169 *recA1 endA1 hsdR17* (rK$^-$, mK$^+$) *phoA supE44* λ– *thi-1 gyrA96 relA1*	D. Cameron
531	HB101/pRK24	F- *mcrB mrr hsdS20*(rB$^-$mB$^-$) *recA13 leuB6 ara-13 proA2 lavYI galK2 xyl-6 mtl-1 rpsL20* carrying pRK24	C. Ellermeier
1663	pMTL-YN1C *spo0A*	https://benchling.com/s/seq-UNkNMhwm7iTM9hUsPVTK?m=slm-ZBEfkup3XNdMoKh1nba2	This study
2336	pMTL-YN1C P*slpA LP::mNeonGreen*	https://benchling.com/s/seq-llMulw067qUImytKppQN?m=slm-WHLlee9qmdhLedLSipHm	This study
2337	pMTL-YN1C P*slpA LP::mScarlet*	https://benchling.com/s/seq-0fLnqXBzAPKKAVxBpJMW?m=slm-BHG1jgTsdYVII9DbIrgY	This study
2426	pMTL-YN1C P*sspB::mScarlet*	https://benchling.com/s/seq-ZXIiLRFuBdZ6WF1vrlhK	This study
2430	pMTL-YN1C P*sspB::mNeonGreen*	https://benchling.com/s/seq-MoclSqPtu7CdxvcVN9sX	This study
2449	pMTLYN3 Δ*tcdR*	https://benchling.com/s/seq-aRlLDyHU9BSUGipQs9yg	This study
2450	pMTLYN1C P*tcdA::mNeonGreen*	https://benchling.com/s/seq-vH5Y4Z7AIW9aVOqEgoT0	This study
2451	pMTLYN1C P*tcdA::mScarlet*	https://benchling.com/s/seq-bs5Ihsmgm78j4Vzrw3uK	This study
2499	pMTLYN1C P*sipL::mScarlet SipL RBS*	https://benchling.com/s/seq-guzj4mJfjx5nsdVzgHL1?m=slm-gWFOorOQFWph1oLQiU2d	This study
2519	pMTL-YNIC *mScarlet LP with SpeI*	https://benchling.com/s/seq-mHd33NoglI7NcPKpOpNb?m=slm-LPl9zquLCvKG6xqxIPCG	This study
2520	pMTL-YN1C *mNeonGreen LP with SpeI*	https://benchling.com/s/seq-UewUwtgn8QYEzfimt63e?m=slm-WEn5M0WVb5vcj3CJq63C	This study
2598	pMTL-YN3 Δ*rstA*	https://benchling.com/s/seq-8SlIH5cOhBsn5fSKrI7T?m=slm-9kf4rykfCHefQBMdc3wL	This study
2966	pMTL-YN1C *tcdR*	https://benchling.com/s/seq-AyR6kox9NT6V8s02pG35?m=slm-8X93KQyyQcD5Y83EFfyX	This study
2968	pMTL-YN1C *rstA*	https://benchling.com/s/seq-pbhznj288AZKN1JTQzOC?m=slm-AmAXNEY1HGCw0fNbe98v	This study
3139	pMTL-YN1C P*cwp2::mScarlet*	https://benchling.com/s/seq-rfzvQvObKO2pwTEM54WH	This study
3141	pMTL-YN1C P*cwp2::mNeonGreen*	https://benchling.com/s/seq-hRC5g9q5YlWemG6lMXi1	This study
3148	pMTL-YN3 P*sipL::mScarlet*	https://benchling.com/s/seq-KobkLvKir7uPbkpV9I4j	This study

Plasmids

pMTL-YN1C	For cloning complementation constructs to be integrated into the *pyrE* locus of 630Δ*erm*Δ*pyrE*	(15)
pMTL-YN3	For cloning allelic exchange constructs to modify 630Δ*erm*Δ*pyrE*	(15)
