# Supplementary material for: Development of a Dual-Fluorescent-Reporter System in Clostridioides difficile Reveals a Division of Labor between Virulence and Transmission Gene Expression
Source: mSphere. 2022 May 31;7(3):e00132-22. doi: 10.1128/msphere.00132-22 (PMC9241537; doi:10.1128/msphere.00132-22)
Supplement: TABLE S2 [file msphere.00132-22-s0010.pdf]

Table S2. Primers used in this study.

#	Primer name	
2088	3' SbfI Δ*sipL* 639 bp gibson	agcaaggcaagaccgatcgggccccctgcaggGTTTGTTAGCTTTTCTCCAATACCTTG
2911	5' NotI P*slpA* Gibson	ggaattagggatgtaataagcggccgcGTTGAGTTCTAAGTTTGCATATACTGG
2912	3' XhoI *mScarlet* Gibson	tgccaagcttgcatgtctgcaggcctcgagTTACTTATATAATTCATCCATTCCTCC
2913	3' XhoI *mNeonGreen* Gibson	gccaagcttgcatgtctgcaggcctcgagTTATTTATATAATTCATCCATACCCATTAC
3014	5' soe P*slpA::mNeonGreen* leader peptide fusion	GGTAATATTAACAATAATTTATTTTCAATGGTAAGTAAAGGAGAAGAAGATAATATG
3015	3' eos P*slpA::mScarlet* leader peptide fusion	CATATTATCTTCTTCTCCTTTACTTACCATTGAAAATAAATTATTGTTAATATTACC
3016	5' soe P*slpA::mScarlet* leader peptide fusion	GGTAATATTAACAATAATTTATTTTCAATGGTATCTAAAGGAGAAGCAGTAATAAAAG
3017	3' eos P*slpA::mScarlet* leader peptide fusion	CTTTTATTACTGCTTCTCCTTTAGATACCATTGAAAATAAATTATTGTTAATATTACC
3066	5' AscI SipL-mNeonGreen 1104	gtcaattgttcaaaaaaataatggcggcgcgccGGAAGGCATACAAAAGCACAGAAAGG
3123	5' NotI P*sspB* YN1C Gibson	CAAGGAATTAGGGATGTAATAAGCGGCCGCAATATTTAATTTATAAGACAATAAC
3124	3' P*sspB* SOE mNeonGreen	TATTATCTTCTTCTCCTTTACTTACCATAGTAAATTTCCTCCTCAAATAATTAG
3125	5' P*sspB* SOE mNeonGreen	CTAATTATTTGAGGAGGAAATTTACTATGGTAAGTAAAGGAGAAGAAGATAATA
3126	3' P*sspB* SOE mScarlet	TTATTACTGCTTCTCCTTTAGATACCATAGTAAATTTCCTCCTCAAATAATTA
3127	5' P*sspB* SOE mScarlet	TAATTATTTGAGGAGGAAATTTACTATGGTATCTAAAGGAGAAGCAGTAATAA
3129	5' NotI SpeI mNeonGreen	aattagggatgtaataagcggccgcACTAGTTGGGAGGAATTTAAGAAATGGTAAG
3130	5' NotI SpeI mScarlet	aattagggatgtaataagcggccgcACTAGTTGGGAGGAATTTAAGAAATGGTATC
3145	5' AscI Δ*tcdR*	gtcaattgttcaaaaaaataatggcggcgcgccgtcatctctatagtaatctagaacttgtttc
3146	5' Δ*tcdR* SOE	ttagcaagaaataactcagtagatgatttggctaaaaatagagcatttaaaaaaataaaa
3147	3' Δ*tcdR* rev eos	ttttatttttttaaatgctctatttttagccaaatcatctactgagttatttcttgctaa
3148	3' SbfI Δ*tcdR*	caaggcaagaccgatcgggccccctgcagggtcaaatctagggtcatttaagttttctc
3152	5' NotI *tcdA* promoter mNeonGreen Gibson	ggatgtaataagcggccgcAAAAGCTAGCCGTGATGAAGGAC
3153	3' SpeI *tcdA* promoter mNeonGreen Gibson	CTTACCATTTCTTAAATTCCTCCCAACTAGTgtattattatttttgataataaatcc
3156	5' NotI *tcdA* promoter mScarlet Gibson	GAGTTCAAGGAATTAGGGATGTAATAAGCGGCCGCaaaagctagccgtgatgaagg
3157	3' SpeI *tcdA* promoter mScarlet Gibson	GATACCATTTCTTAAATTCCTCCCAACTAGTgtattattatttttgataataaatcc
3180	5' NotI *sipL* promoter Gibson	aattagggatgtaataagcggccgcGAGAGACATATAGGAAAGAAAATATTGTTGAAAGC
3221	5' P*sipL::mScarlet* Gibson	GTGTGATATTTTTATAATACTTAAGGAGGTAGACTATGGTATCTAAAGGAGAAGCAGTAA
3222	3' P*sipL::mScarlet* Gibson	TTACTGCTTCTCCTTTAGATACCATAGTCTACCTCCTTAAGTATTATAAAAATATCACAC
3355	5' AscI Δ*rstA*	gtcaattgttcaaaaaaataatggcggcgcgccggggttcctaagaacctctaatctat
3356	5' Δ*rstA* SOE	attttgagcttaggagaaaagattaaaaagtcaaaagaaaaagagttagaatattatcaa
3357	3' Δ*rstA* rev eos	ttgataatattctaactctttttcttttgactttttaatcttttctcctaagctcaaaat
3358	3' SbfI Δ*rstA*	agcaaggcaagaccgatcgggccccctgcaggcatttgattcattacttaatcctttct
3649	3' *sipL* homology mScarlet EOS	ATACTTAATTACTTTAAATTTTTTAATATTATTACTTATATAATTCATCCATTCCTCCTG
3650	5' *sipL* homology mScarlet SOE	CAGGAGGAATGGATGAATTATATAAGTAATAATATTAAAAAATTTAAAGTAATTAAGTAT
3703	5' NotI *tcdR* complementation	gaattagggatgtaataagcggccgccatttgattaaaaataacaaaatattaaataatt
3704	3' XhoI *tcdR* complementation	gcttgcatgtctgcaggcctcgagttacaagttaaaataattttcatagtcttttttat
3705	5' NotI *rstA* complementation	ggaattagggatgtaataagcggccgcgctcttagtttctttttagatgaatcacttcc
3706	3' XhoI *rstA* complementation	agcttgcatgtctgcaggcctcgagctacattatttctaagtttttgtacataaatacac

3762	5' SipL TAA P*sipL* gibson	ATTTTAGAAAAAAAAGTCGTATTAGTAGATTAAGAGAGACATATAGGAAAGAAAATATTG
3795	3' SipL TAA P*sipL* gibson	CAATATTTTCTTTCCTATATGTCTCTCTTAATCTACTAATACGACTTTTTTTTCTAAAAT
3884	5' NotI *cwp2* Promoter	gggatgtaataagcggccgcttcatgtaatttttattaaatgaaaaataaagtattttga
3885	3' SpeI *cwp2* Promoter	tttcatttcttaaattcctcccaactagtTTTATTATTTTTTCCTTATTTACCAATTATA
3915	5' P*sipL* NotI reporter gibson	gaattagggatgtaataagcggccgcGAGAGACATATAGGAAAGAAAATATTGTTGAAAG
3917	3' SpeI P*sipL* Single RBS	catTTCTTAAATTCCTCCCAACTAGTATTATAAAAATATCACACTATATTTATATTTAAG
gblock 40	P*slpA* with 18aa leader peptide	GTTGAGTTCTAAGTTTGCATATACTGGATTTGGGTTATATATATTACCAGATGGACAAGTG TATGCTACTCAGGAGTTTTTAAATAAGTAAAATTAAATTTTTTAGTTTATTACATTTTAAAA TTTAGGGTATAAAAACTTGTAAACTTGGAGAAAATAATAATTTAAAAAAATAGCTTGCAA AAAGAATAAAAATGGATTATTATAGAGATGTGAGAAATATTAGGAATATATGGATGATTA TTCTATGTACATAATAAAGAGATGTAATTTTAATATAATGTTGGGAGGAATTTAAGAAatgaa agcactcgaaaagagattcaaaggtaatattaacaataatttattttca

Restriction sites are underlined.
